# Unexpected stability of aqueous dispersions of raspberry-like colloids

**DOI:** 10.1038/s41467-018-05560-3

**Published:** 2018-09-06

**Authors:** Yang Lan, Alessio Caciagli, Giulia Guidetti, Ziyi Yu, Ji Liu, Villads E. Johansen, Marlous Kamp, Chris Abell, Silvia Vignolini, Oren A. Scherman, Erika Eiser

**Affiliations:** 1 Melville Laboratory for Polymer Synthesis, Department of Chemistry, University of Cambridge, Cambridge, CB2 1EW UK; 2 Department of Chemistry, University of Cambridge, Cambridge, CB2 1EW UK; 3 Cavendish Laboratory, University of Cambridge, Cambridge, CB3 0HE UK

## Abstract

Aqueous colloidal suspensions, both man-made and natural, are part of our everyday life. The applicability of colloidal suspensions, however, is limited by the range of conditions over which they are stable. Here we report a novel type of highly monodisperse raspberry-like colloids, which are prepared in a single-step synthesis that relies on simultaneous dispersion and emulsion polymerisation. The resulting raspberry colloids behave almost like hard spheres. In aqueous solutions, such prepared raspberries show unexpected stability against aggregation over large variations of added salt concentrations without addition of stabilisers. We present simple Derjaguin–Landau–Verwey–Overbeek (DLVO) calculations performed on raspberry-like and smooth colloids showing that this stability results from our raspberries’ unique morphology, which extends our understanding of colloidal stability against salting. Further, the raspberries’ stability facilitates the formation of superspheres and thin films in which the raspberry colloids self-assemble into hexagonally close-packed photonic crystals with exquisite reproducibility.

## Abstract (web summary)

The ability to stabilise colloidal suspensions in solution against salt-induced aggregation is critical to many industrial applications, but it remains challenging at high salt concentration. To overcome this problem, Lan et al. introduce a raspberry-like colloidal particle with controllable morphology.

## Introduction

The ability to disperse charged colloids in water is generally determined by the interplay between attractive van der Waals (vdW) and repulsive Coulomb interactions^1–4^. This balance can be shifted easily from long-ranged repulsion to irreversible aggregation between colloids upon variation of external conditions such as adding varying salts. Polymeric and other steric stabilisation schemes have been established to adjust the overall interactions between colloids to maintain stability in varying conditions; however, this often involves multi-step efforts and also changes the nature of the colloidal system^5^.

The hierarchical architecture of raspberry-like particles, resembling that of lotus-leave surfaces^6^, makes them excellent materials for superhydrophobic coatings^7,8^, water purification^9^ and catalytic applications^10–12^. Further, colloids with similar structure to raspberry particles show enhanced stability against depletion flocculation^13–15^.

Here we show that the unique structure of the raspberry-like colloids (raspberries for short hereafter) presented here also provides excellent stability for their self-assembly into superstructures as well as against salt-induced colloidal aggregation in the absence of any stabiliser. After introducing our one-step synthesis method for obtaining monodisperse, charged raspberries, we demonstrate how their stability against aggregation allows us to form superspheres from emulsion droplets and highly crystalline dry films with photonic properties. Detailed calculations on the interactions between the raspberries and equivalent smooth colloids presented here then explain that it is the particles’ roughness that leads to the unusual stability against aggregation in aqueous suspensions.

## Results

### Synthesis of raspberry colloids

**Fig. 1:**
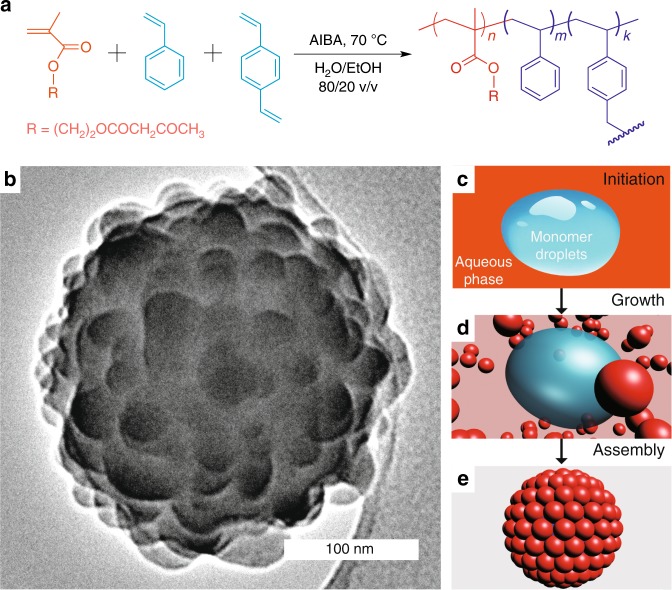
One-step synthesis of raspberry colloids. **a** 2.0, 20.0 and 1.0 mmol of acrylate monomer (AM), styrene (St) and divinylbenzene (DVB) are polymerised in 50 mL of a water–ethanol mixture (80/20 v/v), containing 0.2 mmol 2,2′-azobis(2-methyl-propionamide) dihydrochloride (AIBA) as initiator at 70 °C. Raspberry particles are formed as cross-linked hybrid polymers with polyacrylate (PA) and polystyrene (PS) sections. **b** TEM image of the raspberry particle. **c**–**e** Mechanism of the one-step polymerisation approach for raspberry particles. AM-rich solution and St-rich heterogeneous monomer droplets coexist in the continuous phase at the beginning (**c**). Upon initiation, nanoscale PA-rich particles (created in dispersion polymerisation of dissolved monomers) and microscale PS-rich particles (resulting from soap-free emulsion polymerisation of heterogeneous monomer droplets) form in the same system (**d**), which are brought together in the presence of cross-linking agent of DVB, forming raspberry colloids (**e**). Images are not to scale

We synthesise the raspberry colloids by injecting a ternary mixture of acrylate monomers (AMs), styrene (St) and the cross-linker divinylbenzene (DVB) into a water–ethanol solution (80/20 v/v) containing the initiator 2,2′-azobis(2-methylpropionamide) dihydrochloride (AIBA) at 70 °C. Exploiting the different solubilities of AM and St monomers in water–ethanol mixtures (Supplementary Figs. 1–3; Supplementary Table 1, Supplementary Note 1), we induce simultaneously dispersion polymerisation of the AMs in the continuous phase forming ~45 nm-sized polyacrylate (PA) particles and the soap-free emulsion polymerisation of St-rich emulsion droplets producing roughly 200 nm-sized particles containing mainly polystyrene (PS). The 45 nm PA and 200 nm PS-rich particles then fuse to the final shape of raspberry colloids in the presence of cross-linker DVB (Fig. 1; Supplementary Fig. 4). The three-dimensional (3D)-rendering video (Supplementary Movie 1) obtained from transmission electron microscopy (TEM) demonstrates the uniformity of the raspberry particles.

**Fig. 2:**
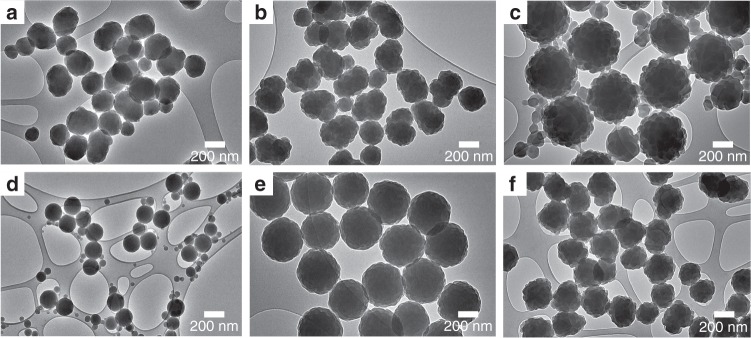
Effect of solvent condition and cross-linker on raspberry-colloid synthesis. TEM images of colloids synthesised in **a** pure water, **b** a 90/10 v/v, and **c** in a 70/30 v/v water/ethanol mixture, respectively, using in all three cases the same composition of AM, St and the cross-linker DVB used for ideal raspberries. The average hydrodynamic diameter of the particles determined by dynamic light scattering (DLS) were 300, 245 and 330 nm, respectively. **d** TEM image of particles synthesised using the ideal 80/20 v/v water/ethanol mixture but omitting DVB. **e**, **f** Colloids synthesised in the same 80/20 v/v mixture but using only St and DVB (**e**) and St, DVB plus half the required amount of AM (**f**), respectively

The solvent conditions and the cross-linker DVB are crucial in the formation of the raspberry shape as demonstrated in Fig. 2 (see below). Using identical concentrations of ingredients as for the perfect raspberry particles but replacing the solvent either by pure water or different water to ethanol ratios produced non-uniform, highly polydisperse particles (Supplementary Note 2). Focussing only on the ‘correct’ 80/20 v/v ratio but removing the cross-linker delivered larger PS-rich colloids coexisting with detached smaller polymethylmethacrylate (PMMA) particles (Supplementary Fig. 5, Supplementary Note 3). Further, while keeping the DVB and St concentrations constant ‘bumpy’ PS particles with a narrow size distribution were produced in the absence of AM monomers, and highly polydisperse raspberry particles formed when only half the AM amount was used (Supplementary Note 4). Using the same method, the AM monomers could easily be replaced by either methyl-, ethyl- or heptafluorobutyl methacrylate and differently sized raspberries could be obtained (Supplementary Fig. 6, Supplementary Note 5). Our synthesis protocol delivers reproducibility ~280 nm large raspberry colloids with a polydispersity index 0.02. After purification, the final raspberry suspensions with a colloidal volume fraction of ~3% have an ionic content of roughly 0.5 mM and an effective zeta potential (ZP) of around +42 mV; the suspensions proved stable for over 6 months showing no colloidal aggregation.

### Formation of superspheres from emulsion droplets

**Fig. 3:**
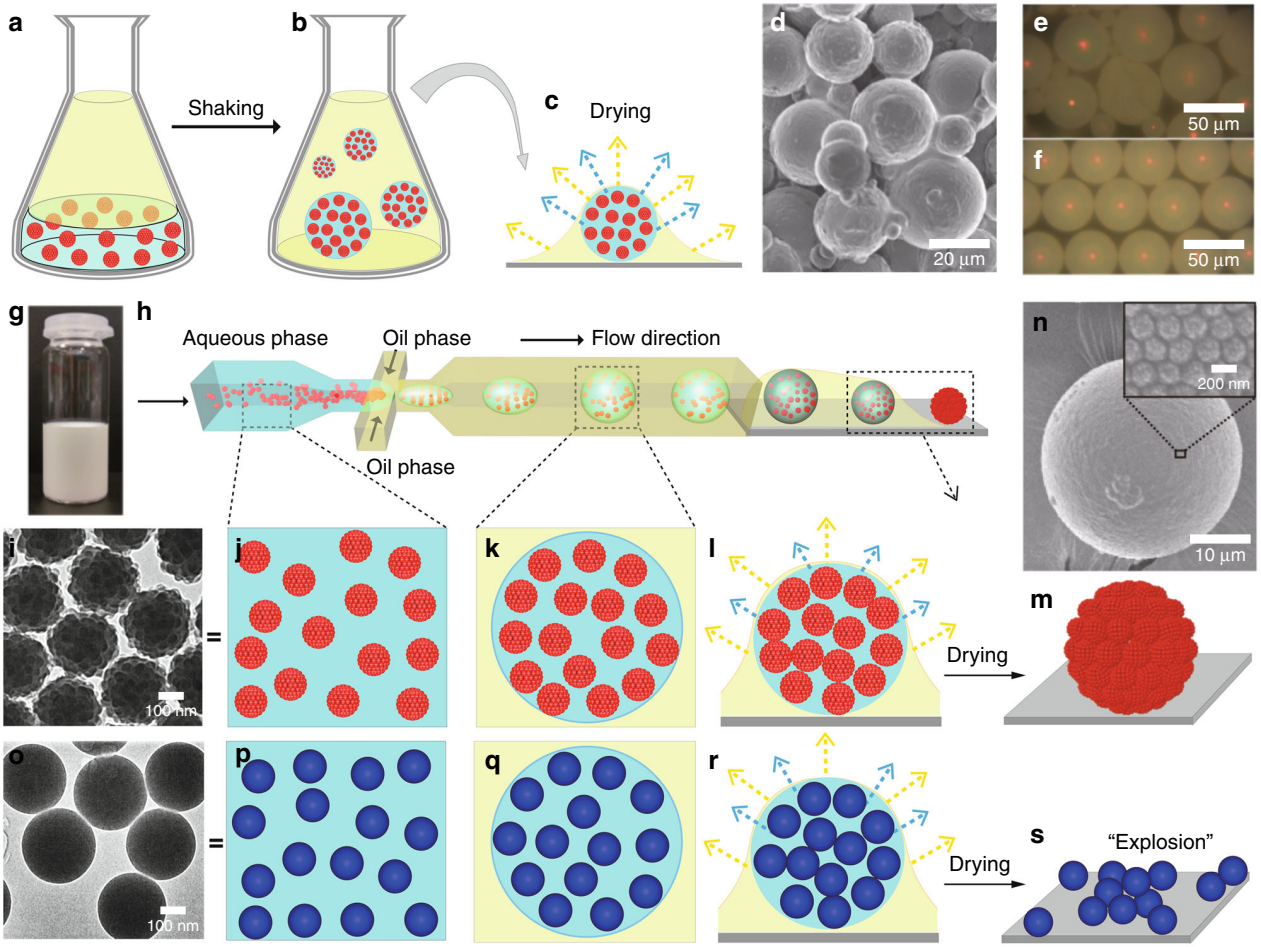
Difference in colloidal stability of emulsion droplets made of either raspberry or smooth particles. **a** A ∼20 w% dispersion of raspberries is mixed in 1:3 parts with fluorinert oil FC40 containing 0.01 w% surfactant FX171 (Sigma Aldrich) (**b**), vigorously mixed (**c**) and then left to dry. **d** SEM image of the resulting dried superspheres. **e**, **f** Microscopic images taken in reflection from superspheres prepared by the manual emulsification process and via microfluidics, respectively. **g** Photograph of the raspberry solution. **h** Schematic of emulsion-droplet preparation of raspberry dispersions via microfluidics. The dispersion (∼20 w%) is injected (flow rate: 100 μL/h) into a device with a T-junction geometry with the same fluorinert oil and surfactant (flow rate: 200 μL/h) budding off the colloid-containing water droplets. The resulting droplets are then dried in air on a glass substrate. **i**–**m** TEM image of the dried raspberries and enlarged schematics of the supersphere-formation process via microfluidics are shown. **n** SEM images of a supersphere made of raspberry particles and a zoomed-in section, showing the colloidal crystallinity at the surface. **o** TEM image of similarly sized smooth PS particles. **p**–**r** Cartoon showing the emulsion-droplet formation containing the smooth PS particles under the same conditions as for raspberries. **s** The droplets that were initially stable in oil suddenly burst in the drying process, leaving the PS particles randomly distributed on the substrate (Supplementary Movie 2)

**Fig. 4:**
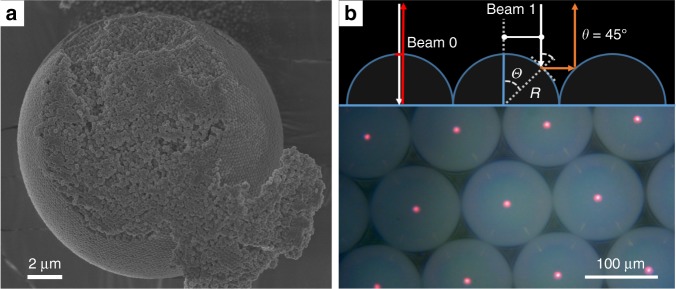
SEM image of a supersphere and Bragg reflection of it. **a** Scanning electron micrograph of a dried supersphere formed from a raspberry dispersion in a microfluidic device as described in Fig. 3. **b** The top image describes the different optical effects produced by densely packed superspheres as observed under the microscope. The red centre spots are caused by specular reflection of non-propagating wavelengths, and the streaks of light are seen where the superspheres touch each other and light is reflected straight back into the microscope. The bottom image is a micrograph obtained in epi-illumination of the superspheres using as small numerical aperture for the illumination (NA = 0.1)

In an attempt to form superspheres with photonic properties, we formed emulsion droplets from raspberry suspension in fluorinert oil (FC-40) by either vigorously shaking the mixture in a flask (Fig. 3a–e) or by using a microfluidic devise (Fig. 3f–m; see below). In both cases, the water-in-oil emulsion droplets were left to dry on glass substrates. Through the evaporation process locally a flow of water towards the droplet surface sets in, bringing the colloids towards it. The surface confinement typically promotes crystallisation, though here in small facets because of the spherical geometry^16^. In a scanning electron microscopy (SEM) image of a dried supersphere that was cut with a scalpel, we counted a packing of several layers at the supersphere’s outer surface, while the interior is rather disordered (Fig. 4a; see below). The surface crystallinity of the superspheres gives rise to the bright red spot due to Bragg reflections on the centre-top of the superspheres observed in optical microscopy, confirming that the ordering is sufficient to produce a coherent scattering response (Figs. 3e, f and 4b)^17^.

Raspberry colloidal dispersions show robust stability during the formation of superspheres irrespective of the preparation method. For comparison, we tested the generality of the supersphere formation using ‘smooth’ colloids purely made of PS with a roughness of ~1 nm, which have a similar size and effective ZP as the raspberry particles (Fig. 3o; Supplementary Note 6). We refer to them as smooth PS (and when relevant PMMA) particles. When the emulsion droplets containing smooth PS colloidal dispersions were left to dry (evaporate) under the same conditions as for the preparation of superspheres containing raspberry particles, the initially spherical emulsion droplets disintegrated into random colloidal debris upon shrinking to a critical size (Fig. 3p–s; Supplementary Movie 2). This disintegration occurred without any exception, irrespective of the emulsification process or addition of salt to prevent strong Coulomb repulsion between the smooth PS particles.

### Stability of raspberry suspensions against salting

**Fig. 5:**
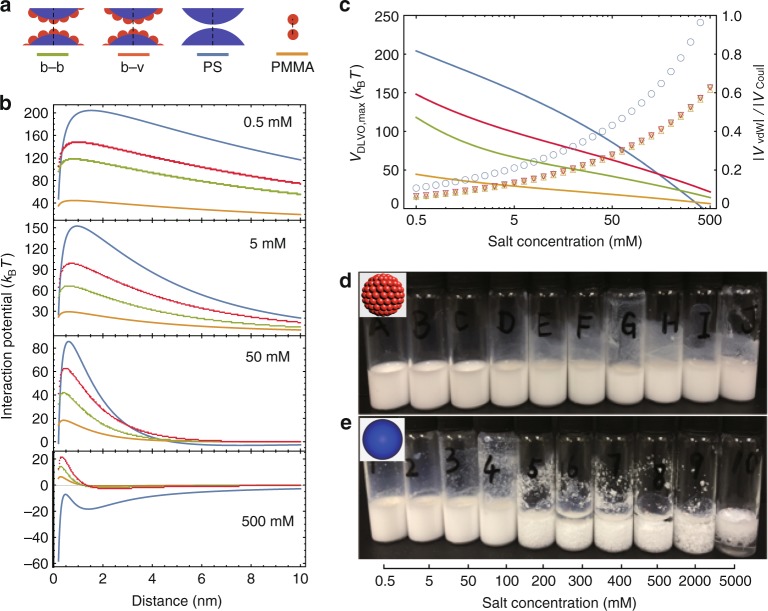
DLVO calculation and colloidal stability in varying salt concentrations. **a** Possible contact geometries between raspberry particles (bump-to-bump (b–b, green lines) and bump-to-valley (b–v, red lines)), smooth PS (blue lines) and nanoscale PMMA particles (yellow lines). **b** Interaction potentials *V*_DLVO_ for raspberry, smooth PS and nanoscale PMMA particles (same colour scheme as in **a**) for 0.5, 5, 50 and 500 mM added NaCl. **c** Primary energy maximum *V*_DLVO,max_ (solid lines) and relative strength of attractive interactions to repulsive forces ($\left| {V_{\mathrm{vdW}}} \right|{\mathrm{/}}\left| {V_{\mathrm{Coul}}} \right|$; symbols) for raspberry particles (b–b (diamonds) and b–v (inverted triangles)), smooth particles (circles) and nanoscale PMMA particles (triangles) against salt concentration. **d**, **e** Photographs of dispersions of raspberry particles and smooth particles, respectively, at different added NaCl concentrations

To understand the difference in stability of the raspberry and smooth PS colloidal suspensions, we estimated the DLVO (Derjaguin–Landau–Verwey–Overbeek) interactions between both types of colloids (Fig. 5; see below and Supplementary Note 7). The total pair-wise interactions are due to the sum of the attractive vdW and repulsive Coulomb double-layer interactions: *V*_DLVO_ = *V*_vdW_ + *V*_Coul_. We modelled a raspberry particle as a PS core (radius *R*_PS_ = 117.5 nm) decorated with PMMA hemispheres of radius *R*_PMMA_ = 22.5 nm closely mimicking the real particles (Fig. 5a; see below). No analytical expression for *V*_vdW_ exists for such complex geometries. Hence we approximate the interactions using the sum of pair-wise *V*_vdW_ between the hemispheres and the core particles for two configurations: bump-to-bump (b–b) and bump-to-valley (b–v) with respect to the ‘hard’ hemispheres, as sketched in Fig. 5a (Supplementary Fig. 8). Details of the calculations are presented in Supplementary Note 7. For comparison, *V*_DLVO_ for the smooth PS (280 nm) and PMMA (45 nm) particles with a ZP of +42 mV (Fig. 5b, Supplementary Fig. 9) were computed and are presented as a function of particle separation for 0.5 mM added NaCl salt, which corresponds to our experimental starting configuration. The DLVO calculations were then repeated with the same settings but for increasing salt concentrations up to 500 mM added NaCl. Application of the DLVO theory for even higher salt concentrations becomes questionable.

In Fig. 5d, e, we demonstrate the stability against salting in a salting series of our raspberry and the similarly sized and charged smooth PS particles. We start with an added salt concentration of 0.5 mM and go up to 5 M. While the raspberry suspensions remain stable even at the highest ionic strengths, the smooth PS particles start to flocculate already above 100 mM.

### Self-assembly of raspberry colloids

**Fig. 6:**
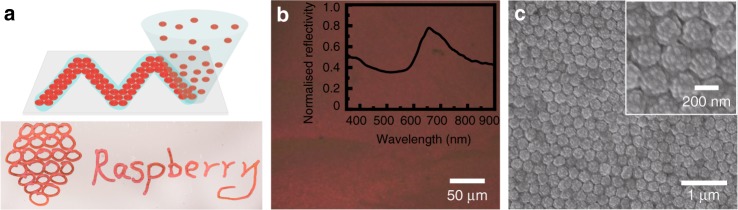
Reflectivity properties of raspberry-particle ink. **a** Cartoon illustrating how raspberry colloidal dispersions (3 w%) can be used as ink on a coverslip and a corresponding sample photographed in reflection at a specific angle. **b** Microscopic image taken in reflective mode of a dried film made of raspberries and the corresponding reflectivity curve (inset). **c** SEM image of self-assembled raspberry particles on a glass substrate

The stability of our raspberry particles allows for spontaneous self-assembly into lattice structures with a strong optical response. When spreading a raspberry suspension on a clean glass slide and letting it dry, we observe the formation of a dry film with highly ordered regions at the film–air interface, as demonstrated in the SEM image in Fig. 6c (see below). A clear hexagonal packing typical for face-centred cubic structures is visible, in agreement with the structure obtained at the surface of the photonic superspheres. Reflectivity measurements show a clear reflectance peak at around 660 nm due to Bragg reflection. These raspberry suspensions can also be used directly as ‘photonic inks’ whose colouration depends on the size of the raspberry particles (Fig. 6a; Supplementary Fig. 7, Supplementary Note 8). Passing a 3 vol.% suspension of raspberry particles through a nozzle, it is possible to obtain an ordered 3D photonic crystals^18^ when writing on glass surfaces like a ‘photonic pen’. In contrast to conventional opals obtained with smooth spheres, meniscus effects during solvent evaporation play a marginal role in the assembly of our raspberries into opalescent structures and represent a much more robust system.

## Discussion

We demonstrate that raspberry particles, synthesised in a single step, display unusual colloidal stability against large variations of salt concentration due to their unique morphology. This polymerisation process is carried out in a single step making it more efficient and sustainable than traditional multi-step methods^15,19–24^. We propose that this one-step synthesis crucially depends on the solvent conditions and the exact ratio of St to AM monomers. In particular, the presence of ethanol as co-solvent increases the solubility of both monomers in the continuous phase leading to a dispersion polymerisation of the AMs into AM-rich chains that eventually phase separate out of solution and form small PMMA particles. Simultaneously emulsion polymerisation of random St-rich co-polymers sets in and larger PS particles form. Our hypothesis that they form simultaneously before assembling into the raspberry structure is supported by the fact that in the absence of the cross-linker separate PMMA and PS particles were formed (Fig. 2d and Supplementary Fig. 5). This observation also suggests that Marangoni effects are not relevant here. Interestingly, when using the 80/20 v/v water to ethanol ratio with the initiator and the St only we obtained monodisperse PS particles as expected but with considerable roughness that is larger than that of the smooth ones produced in pure water and smaller than that of the real raspberries. Yet, in all cases we measured a comparable ZP. We hypothesise that these bumps in pure St systems are due to the higher amount of St monomers dispersed in the ethanol–water mixture that will condense onto the PS core particles due to the slow diffusion of the St monomers towards the emulsion droplets. This roughness difference plays an important role in the colloids’ stability against salting as explained later. It is also noteworthy that using either a different solvent ratio or monomer ratio will lead to highly polydisperse, non-spherical raspberry colloids, emphasising the uniqueness of our one-step synthesis.

Compared to charged, smooth colloids dispersed in a non-refractive index matched solvent, the raspberry particles presented here show a quasi hard-sphere behaviour at both low and high ionic strength. In particular, at high added salt when the Coulomb repulsions are strongly screened smooth colloids typically show irreversible aggregation due to vdW forces. This stability against flocculation also results in our raspberries’ excellent ability to self-assemble into complex superstructures under a wide range of conditions. Furthermore, despite their obvious ‘roughness’, raspberry colloids form smooth crystalline films when dried on flat surfaces (Fig. 6). This crystallinity is also observed on the outer layer of superspheres formed by emulsifying raspberry-colloid suspensions that were subsequently dried. However, the cross-section of such superspheres (Fig. 4) shows that the inside is more disordered, which is simply due to the continuous volume change in the droplet during evaporation forcing the raspberry particles to constantly rearrange and also because a spherical geometry does not support long-range crystallinity. But because our raspberry colloids do behave almost like hard spheres and because they are highly monodispersed despite their ‘roughness’, they do crystallise into a face-centered cubic symmetry at the air–colloid film interface, giving rise to a strong Bragg reflection at around 660 nm, making them appear brilliantly red under some angles (Figs. 2, 6).

As our one-step synthesis of raspberry colloids is completely soap-free, it is straight forward to use the classical DLVO theory to estimate the pair-wise interactions between them. For simplicity, we assumed a quasi-two-dimensional calculation, though taking the full vdW and Coulomb interactions of the PS cores and PMMA hemispheres into account, as detailed in Supplementary Note 7. We computed the separate contributions and their sum and plot them in Fig. 5 for raspberries in bump-to-bump (b–b) and bump-to-valey (b–v) configuration, the 280 nm large smooth PS particles as well as for smooth PMMA particles of size equivalent to that of the ‘bumps’ on the raspberries. For all salt concentrations, we find that the b–v configuration is energetically more stable than the b–b configuration. At low salt concentration (0.5 mM), we find that *V*_DLVO_ for raspberry colloids, smooth PS and PMMA colloids are all dominated by double-layer repulsions: ~40*k*_B_*T* between the small PMMA colloids, and ~200*k*_B_*T* between the smooth PS colloids; *k*_B_ is Boltzmann’s constant and *T* is here room temperature. For both the raspberry configurations, the primary maximum in *V*_DLVO_ lies in between the smooth PS and the smaller PMMA colloids, in agreement with previous reports on particles with large roughness^25^ (Supplementary Figs. 10, 11, Supplementary Note 7). The DLVO results show that, at low salinity, the smooth PS colloids are subject to much stronger repulsive forces than the raspberry colloids (Fig. 5b). This is because the raspberry particles, on account of their roughness, present an effective interaction plane at larger distances than the smooth particles, resulting in a much smaller overall repulsion (Supplementary Note 7). However, at high salt concentrations (e.g. 500 mM) the interactions between smooth PS colloids become purely attractive, whereas the raspberry and the small PMMA colloids still experience substantial stabilisation (Fig. 5b, Supplementary Note 7). The key point in this case is that, while the range of the Coulomb repulsions becomes shorter with increasing added salt concentrations, the range of *V*_DLVO_ does not change much. However the raspberries’ specific morphology presents a lower effective volume available for the vdW interactions than the smooth PS colloids (Supplementary Note 7). As a consequence, the attraction–repulsion ratio $\left( {\left| {V_{{\mathrm{vdW}}}} \right|{\mathrm{/}}\left| {V_{{\mathrm{Coul}}}} \right|} \right)$ for the raspberry particles decays slower with increasing salt concentration than for the smooth PS colloids, resulting in their stability against salting (Fig. 5c). Varying the bump size between 20 and 60 nm (lower and upper limits for the model validity; Supplementary Fig. 12), our calculations predict that raspberry particles with larger bumps tend to display larger overall stability at all salt concentrations. This suggests that the morphology of the raspberry surface plays an important role in the colloidal stability. Indeed, experiments on pure PS colloids prepared in a 80/20 v/v mixture, which display some roughness, remain stable against salting up to about 500 mM of added NaCl (Supplementary Fig. 13). Our model also predicts that raspberry particles remain dispersed at high salt concentrations for ZPs >20 mV (Supplementary Fig. 14). This limit could be further lowered by reducing the attractive vdW forces (e.g. by replacing the core material with one exhibiting a smaller Hamaker constant). However, in the limit of very large bums we return to the smooth colloid approximation and stability breaks down again at lower added salt concentrations. We also note that aqueous suspensions containing other ‘bump’ materials display enhanced stability against salting out as well (Supplementary Fig. 15).

These findings allow us to understand qualitatively why bulk suspensions of smooth PS colloids become unstable at high salt concentrations, yet disintegrate when confined to a shrinking aqueous droplet (Fig. 3o–s). As the emulsion droplets shrink due to evaporation, their effective salt concentration increases, thus progressively decreasing the overall Debye screening length. At the same time, the total charge content is not changing. Following the arguments by Rayleigh when a droplet with a given charge content shrinks below a critical size, the surface energy holding the droplet in its spherical shape becomes comparable to the Coulomb repulsion^26^. Hence, any surface fluctuation can lead to a so-called Coulomb explosion. The aggregation of the smooth PS colloids at high salt is simply due to the fact that the Coulomb stabilisation breaks down at an added salt concentration between 50 and 500 mM, as is demonstrated in the salting-out experiments (Fig. 5). Clearly, the smooth PS particle dispersions start to show irreversible flocculation above 100 mM added salt. In stark contrast, the raspberry particles remain perfectly dispersed in water with 5 M NaCl (about 10 times higher than the salt concentration in seawater), which is consistent with the trend predicted by the DLVO calculations. Similar behaviour was observed using KCl and with several divalent ions (Na_2_SO_4_ and CaCl_2_) (Supplementary Fig. 13, Supplementary Note 9). At the best of our knowledge, such a stability in high ionic-strength solutions has not been observed previously.

For salt concentrations <100 mM, the repulsion between the smooth PS colloids is much stronger than that between raspberry colloids. As described above, such repulsion is likely to destabilise small aqueous droplets containing many smooth PS colloids (Fig. 3o–s) leading to a Rayleigh instability^26^. Under the same conditions, aqueous droplets containing raspberry colloids do not disintegrate (Fig. 3i–n) because the repulsive interactions between the raspberry particles are much weaker than between smooth PS particles (Fig. 5). We have not attempted to develop a quantitative theory for the fragmentation of droplets containing smooth PS or raspberry colloids here. However, the instability can be circumvented by adding surfactants or other stabilisers^16,17^.

Although DLVO theory for low ionic strength for small spherical particles and at large separation distances is approximate^27^, it provides an explanation for the high stability of our raspberry particles against salt-induced flocculation (Fig. 5d) as well as the high stability during their self-assembly into superstructures (Fig. 3m). It also explains why their *V*_DLVO_ maximum is similar to that of the small PMMA particles. Indeed, sufficient roughness provided by the small colloids is crucial for colloidal stability, which is further supported by enhanced stability of pure but rough PS particles (Supplementary Fig. 13). To this end, theoretical work in conjunction with surface force measurements suggests that at very high ionic strengths DLVO needs to be replaced by new scaling arguments that show that the range of a revised screening length increases again^28,29^. This finding may be of great importance in the development of new super-capacitors. Our raspberry colloids thus offer a broader perspective on the role of particle morphology in colloidal stability and could be readily exploited in applications, such as drilling muds, sustainable paints, food products, drug delivery that demands colloidal stability in varying salt concentrations and colloidal volume fractions or as photonic ink for secure printing and responsive coatings. The morphology-induced stability may also help us to understand phenomena in many systems observed in nature, where hierarchical roughness is observed and might help in preventing, for instance, pathogens sticking to biological surfaces in crowded environments.

## Methods

### Materials

All starting materials were purchased from Alfa Aesar and Sigma Aldrich and used as received unless stated otherwise.

### Preparation of raspberry colloids

A monomer mixture of St (2.08 g, 20.0 mmol), DVB (130.0 mg, 1.0 mmol) and 2-(methacryloyloxy) ethyl acetoacetate (AM, 428.0 mg, 2.0 mmol) was added to a 50 mL water/ethanol mixture (20/80 v/v). Then the initiator of AIBA (54 mg, 0.2 mmol) was added to the mixture and then flushed with nitrogen for 1 h before elevating the temperature. The nitrogen blanket was maintained throughout the polymerisation. The reaction was then left stirring at 70 °C for 24 h. The product was purified by dialysis against deionised water. For Raspberry-MMA, methyl methacrylate (MMA; 208.0 mg, 2.0 mmol) was used instead of AM under the same conditions. For Raspberry-EMA, ethyl methacrylate (EMA; 229.0 mg, 2.0 mmol) was used instead of AM under the same conditions.

### Self-assembly of raspberry colloids in emulsion droplets

The microfluidic device for generating sub-millimetre-sized colloidal superspheres was fabricated by inserting a capillary tube with a 27-G needle into a polydimethylsiloxane (PDMS) tube, which was fixed by ethyl acyanoacrylate instantaneous adhesive. The discontinuous (aqueous phase of the raspberry colloids) and continuous (fluorinert oil FC40 with 0.01 w% FX171) phases were injected into the capillary tube and the PDMS tube, respectively. The flow velocities of the two phases were adjusted by syringe pumps independently: The flow rate for the aqueous phase was 100 μL/h and that for the oil phase was 200 μL/h. The emulsion droplets containing raspberry colloids were collected on a glass substrate. After water evaporation, the superspheres were finally obtained.

### Letter writing using the dispersion of raspberry colloids

To write colloidal photonic crystal script on a dry glass substrate (microscopic slide), a dispersion of raspberry colloids (3 w%) was loaded into a 1 mL syringe. The syringe with a 25-G needle was mounted on a syringe pump and fitted with polyethylene tubing, while the other end of the tubing was inserted into a 27-G plain end needle as a nozzle. The flow rate of the dispersion was set to 500 μL/h. When the raspberry colloids flowed to the 27-G needle end, the needle was held by a tweezer and the letters were written by hand.

### Instrumentation

Attenuated total reflection (ATR) Fourier transform infrared (FT-IR) spectroscopy was performed using a Perkin-Elmer Spectrum 100 series FT-IR spectrometer equipped with a universal ATR sampling accessory. Ultraviolet–visible (UV-vis) studies were performed on a Varian Cary 4000 UV-vis spectrophotometer. TEM characterisation was carried out by a FEI Philips Tecnai 20 TEM under an accelerating voltage of 200 kV. Samples were prepared by applying one drop of the dispersion onto a holey R carbon-coated copper TEM grid (400 mesh) drying overnight. DLS and ZP measurements were performed on a Malvern Zeta-sizer NS90 instrument.

## Electronic supplementary material

Supplementary Information

Description of Additional Supplementary Files

Supplementary Movie 1

Supplementary Movie 2

## Data Availability

The data sets that support the findings of this study are available from the corresponding authors on reasonable request.
