# Supplementary material for: Unexpected stability of aqueous dispersions of raspberry-like colloids
Source: Nat Commun. 2018 Sep 6;9:3614. doi: 10.1038/s41467-018-05560-3 (PMC6127241; doi:10.1038/s41467-018-05560-3)
Supplement: Supplementary file 1 — Supplementary Information [file 41467_2018_5560_MOESM1_ESM.pdf]

Unexpected stability of aqueous dispersions of raspberry-like colloids – Supporting Information

Lan et al.

Contents

1 Supplementary Figures **3**

1.1 UV-*vis* absorption and calibration curve of monomers 3
1.2 UV-*vis* absorption of dissolved monomers 4
1.3 Photo of oil droplets in the continuous phase 4
1.4 FTIR spectrum and hydrodynamic size-distribution of the raspberries 5
1.5 Effect of the cross-linking agent 6
1.6 TEM images of other types of raspberry colloids 7
1.7 Structural color 7
1.8 Raspberry colloids configurations 8
1.9 DLVO curves of single PS and PMMA particles 8
1.10 Potential curves for the b-b, b-v, smooth PS and PMMA particles at different electrolyte concentrations 9
1.11 Primary energy maximum in V_{DLVO} as function of added NaCl concentration 10
1.12 Influence of bump-size on the colloidal stability 11
1.13 Effect of different salts 12
1.14 Influence of zeta-potential on the colloidal stability 13
1.15 Colloidal stability of the other raspberry colloids 14

2 Supplementary Tables **15**

2.1 Table of the solubility of monomers 15
2.2 Table of the numerical coefficients 15

3 Supplementary Notes **16**

3.1 Supplementary Note 1: Solubility of monomers 16
3.2 Supplementary Note 2: Effect of the continuous phase 16
3.3 Supplementary Note 3: Effect of the cross-linking agent 17
3.4 Supplementary Note 4: Effect of AM monomer 17
3.5 Supplementary Note 5: Other types of raspberry colloids 17

3.6 Supplementary Note 6: Synthesis of PS particles 18
3.7 Supplementary Note 7: DLVO calculations 18
3.7.1 Model definition 18
3.7.2 Model Equations 18
3.7.3 DLVO curves of single PS and PMMA particles 19
3.7.4 Raspberry colloids configurations 20
3.7.5 Results of DLVO calculations 20
3.7.6 Influence of bump-size on the colloidal stability 21
3.7.7 Influence of zeta-potential on the colloidal stability 22
3.7.8 Colloidal stability of the other raspberry colloids 22
3.8 Supplementary Note 8: Structural color 22
3.9 Supplementary Note 9: Effect of different salts on the stability of raspberry dispersion . . 23

1 Supplementary Figures

1.1 UV-*vis* absorption and calibration curve of monomers

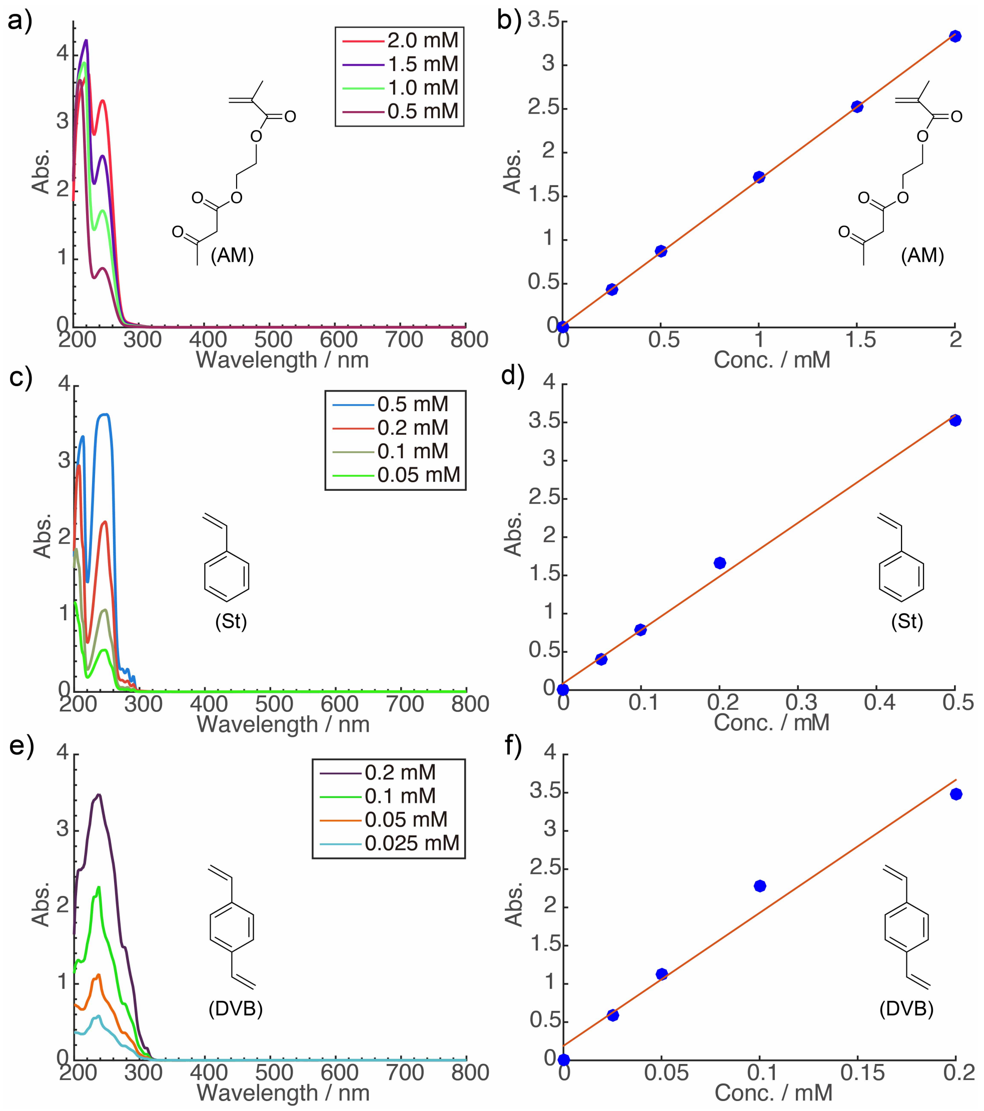

Supplementary Figure 1: Changes in the absorption spectra of different **a,** AM, **c,** St and **e,** DVB monomer concentrations in ethanol, respectively. Calibration curves were obtained using a linear fit to the adsorption maxima for **b,** AM at 244 nm, **d,** St at 254 nm and **f,** DVB at 238 nm.

1.2 UV-*vis* absorption of dissolved monomers

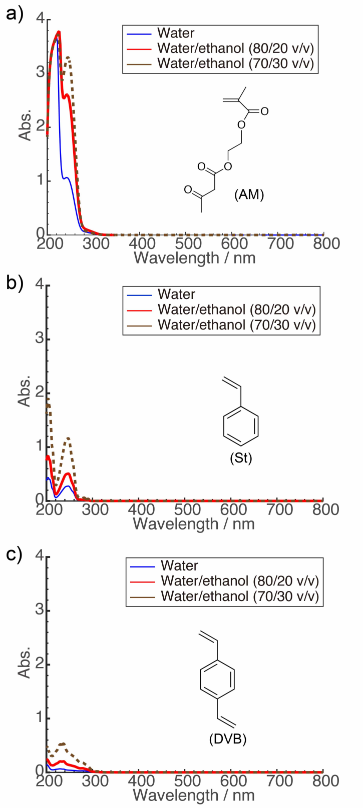

Supplementary Figure 2: UV-vis absorption spectra of water, 80/20 v/v, and 70/30 v/v water-ethanol mixtures saturated in **a,** AM, **b,** St and **c,** DVB monomers, respectively. The saturated solutions were all diluted 100 times before the UV-vis measurements.

1.3 Photo of oil droplets in the continuous phase

A first step in the emulsion polymerisation in which we form the raspberries is to inject a separately formed mixture of St, AM and DVB into the water-ethanol mixture as it is been stirred at 1000 RPM. Large styrene-rich emulsion droplets form, rendering the solution white. The emulsions' typical appearance is shown in Supplementary Figure 3.

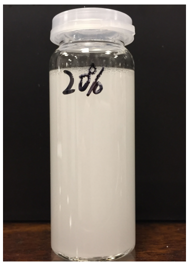

Supplementary Figure 3: Photo of a vigorously stirred emulsion of St (1.04 g, 10.0 mmol), DVB (65.0 mg, 0.5 mmol) and AM (214.0 mg, 1.0 mmol) in 20 mL of water/ethanol mixture (80/20 v/v).

1.4 FTIR spectrum and hydrodynamic size-distribution of the raspberries

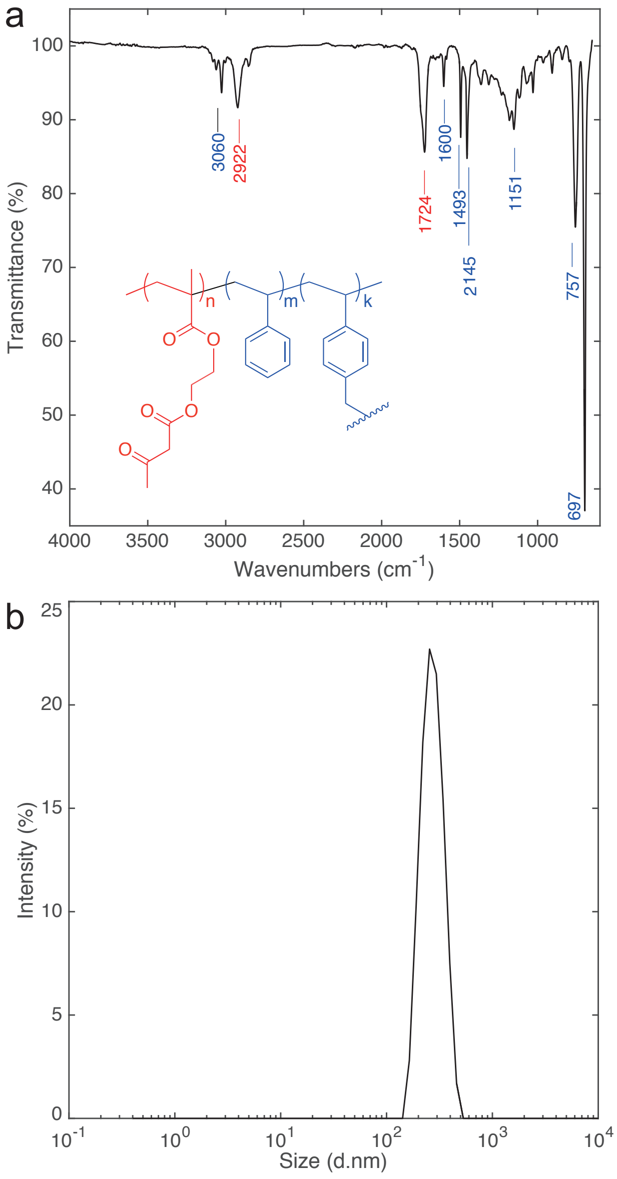

Supplementary Figure 4: a, Fourier transform infrared spectroscopy (FTIR) spectra of the raspberry colloids. It confirms that the raspberry colloids are made of random-block copolymers of polystyrene (PS) and polyacrylates (PA).**b,** Size distribution of the raspberries dispersed in deioninsed water, measured using dynamic light scattering (DLS).

1.5 Effect of the cross-linking agent

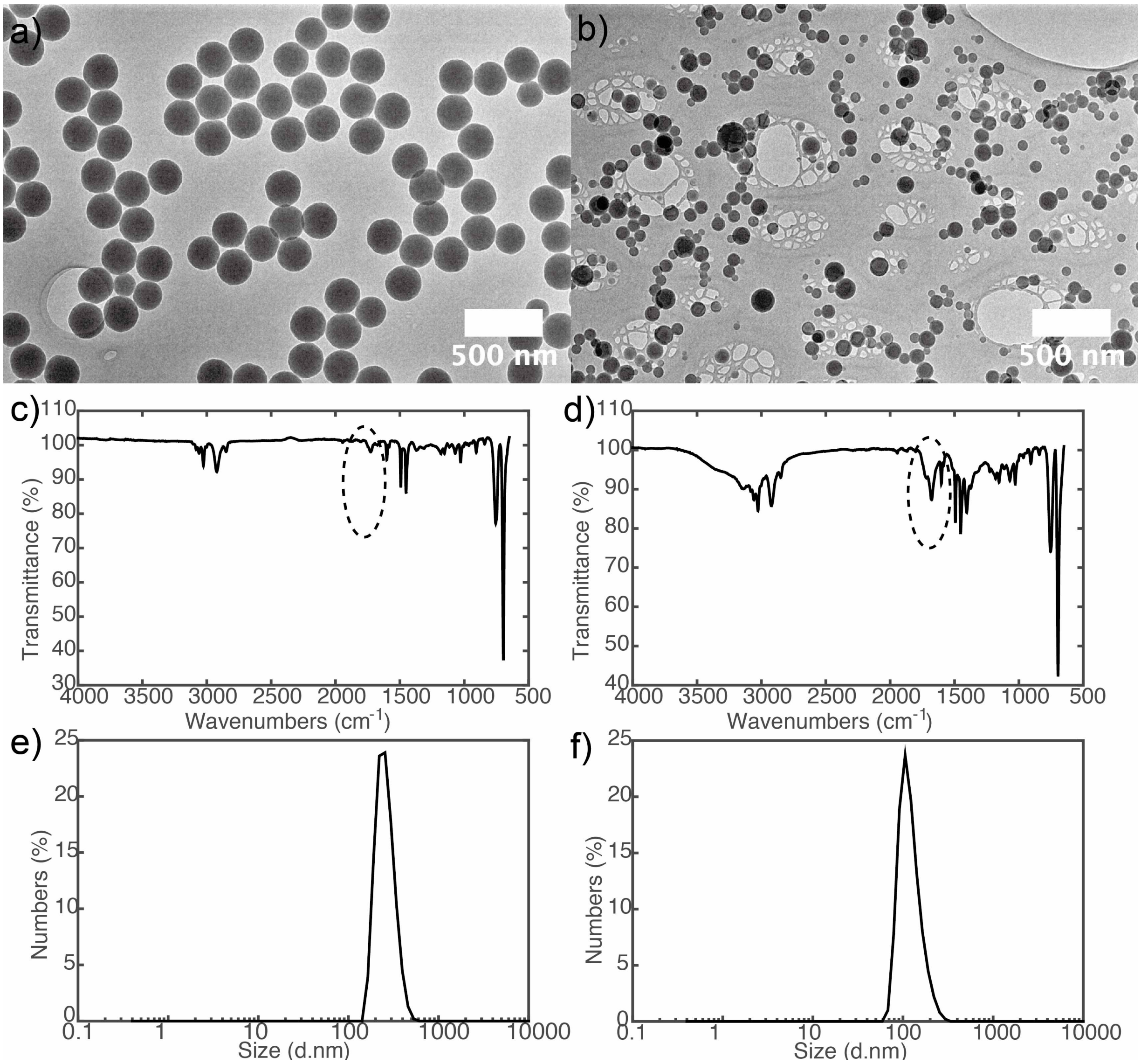

Supplementary Figure 5: TEM images of **a,** bigger colloidal particles and **b,** smaller colloidal particles separated from the control experiment synthesising raspberries in a 80/20 v/v water-ethanol mixture, in which the cross-linker DVB was omitted. Corresponding FTIR spectra of **c,** bigger colloidal particles and **d,** smaller colloidal particles. Hydrodynamic diameter distribution of **e,** bigger colloidal particles and **f,** smaller colloidal particles measured by DLS in water.

1.6 TEM images of other types of raspberry colloids

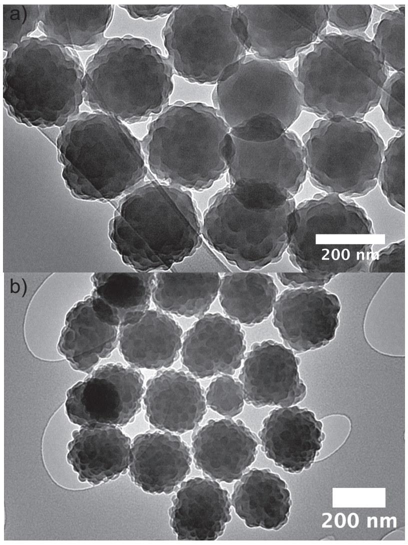

Supplementary Figure 6: TEM images of two different raspberry colloids prepared using a) methyl methacrylate and b) ethyl methacrylate instead of AM.

1.7 Structural color

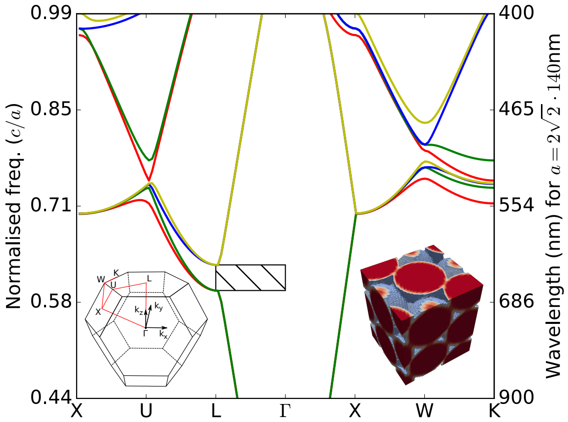

Supplementary Figure 7: Band diagram for FCC packed raspberry colloids. The hatched area indicates a partial band-gap (stop-band) when seen from above. The right axis indicates the wavelengths with which the band-gap will occur for particles of radius $a = 143$ nm, showing a reflection peak between 650 and 700 nm, which corresponds to the measured reflectivity curve in Fig. 4b. The speed of light is denominated c; the left inset shows the labelling of the crystal orientations and the right shows a visualisation of the FCC packed raspberry colloids approximated as spheres.

1.8 Raspberry colloids configurations

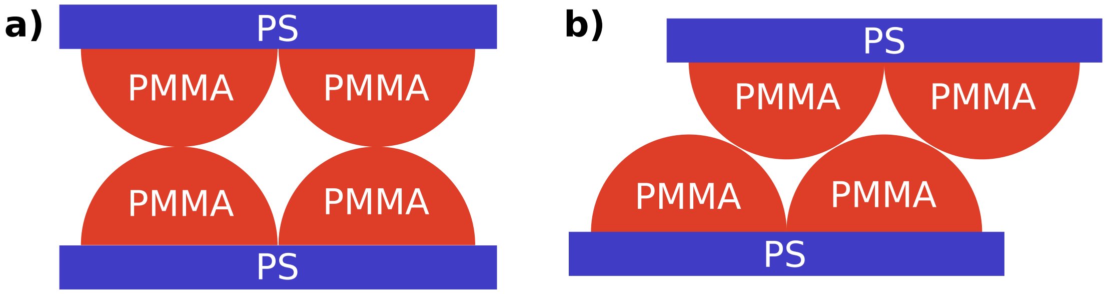

Supplementary Figure 8: Models of raspberry colloids at closest contact in the **a,** bump-to-bump (b-b) and **b,** bump-to-valley (b-v) configuration.

1.9 DLVO curves of single PS and PMMA particles

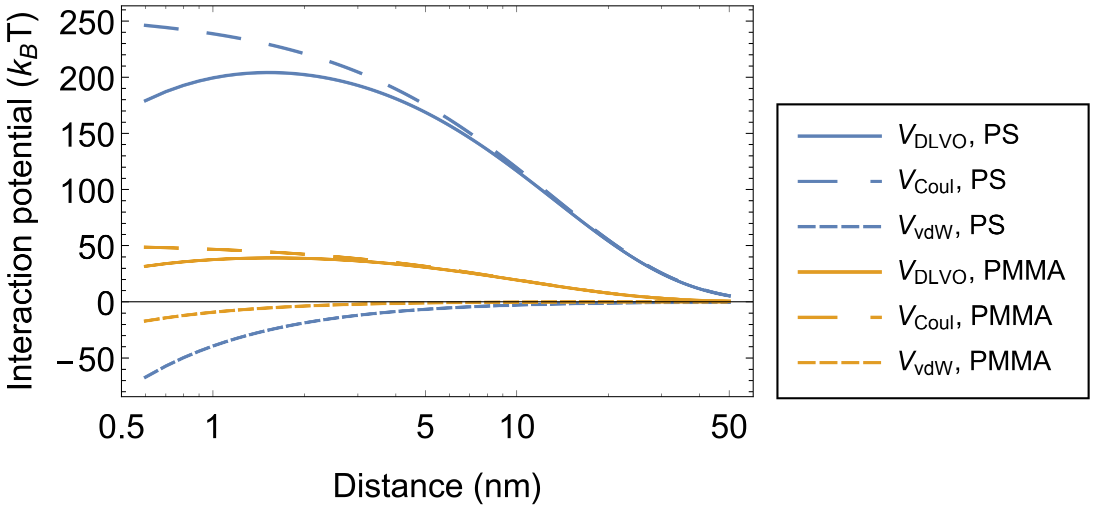

Supplementary Figure 9: DLVO potential curves (solid lines) for a pair of PS spheres (blue) and a pair of PMMA spheres (yellow), with the separate contributions V_{Coul} and V_{vdW} (dashed curves) in a $c = 0.5$ *mM* added NaCl solution. The x-axis is logarithmic for ease of view.

1.10 Potential curves for the b-b, b-v, smooth PS and PMMA particles at different electrolyte concentrations

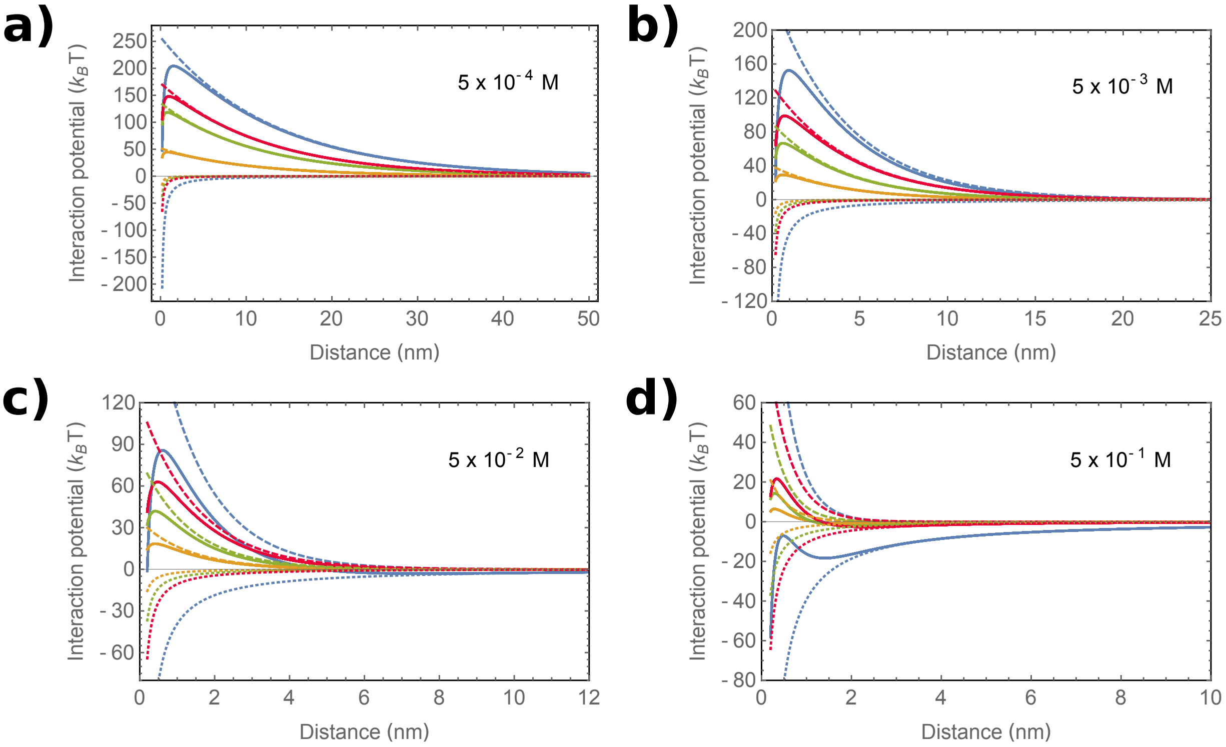

Supplementary Figure 10: Interaction potentials V_{DLVO} (solid lines) for raspberry colloids (b-b, green and b-v, red), smooth PS particles (blue) and nanoscale PMMA particles (yellow) at different salt concentrations of **a,** 0.0005 M, **b,** 0.005 M, **c)** 0.05 M and **d)** 0.5 M added monovalent salts. The dotted and dashed lines are the separate V_{vdW} and V_{Coul} contributions.

1.11 Primary energy maximum in V_{DLVO} as function of added NaCl concentration

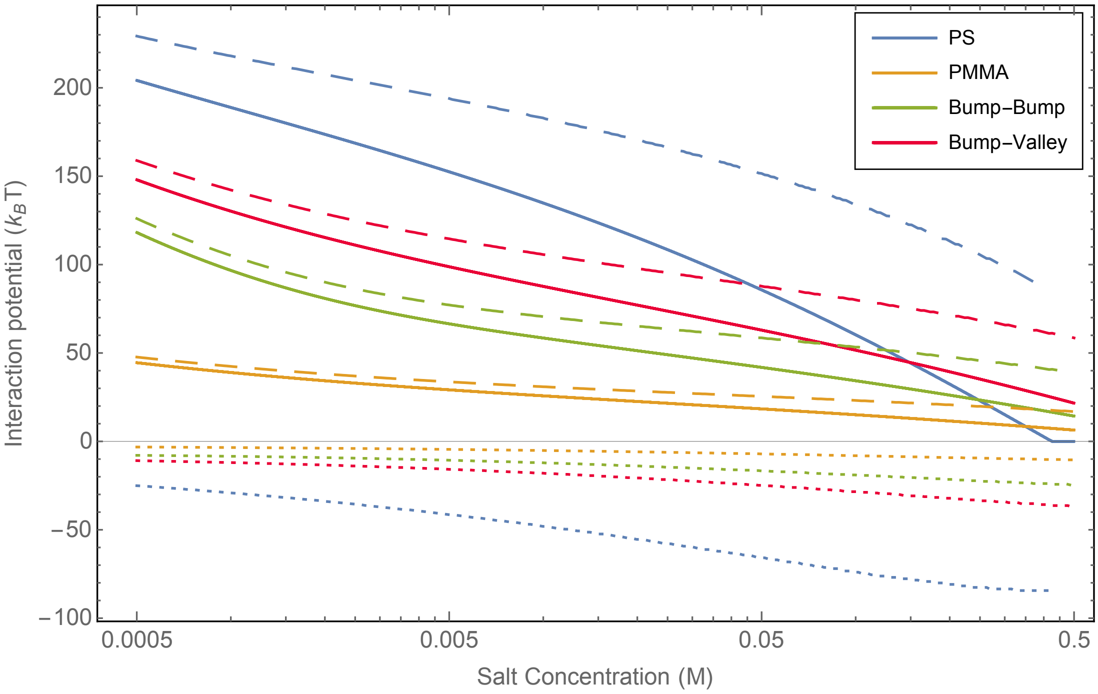

Supplementary Figure 11: Primary energy maximum in V_{DLVO} as function of added NaCl concentration (solid lines), and the contributions from the attractive (V_{vdW}; dotted lines) and repulsive (V_{Coul}; dashed lines) interactions measured at this maximum for raspberry colloids (b-b, green and b-v, red), smooth PS particles (blue) and nanoscale PMMA particles (yellow) against salt concentration.

1.12 Influence of bump-size on the colloidal stability

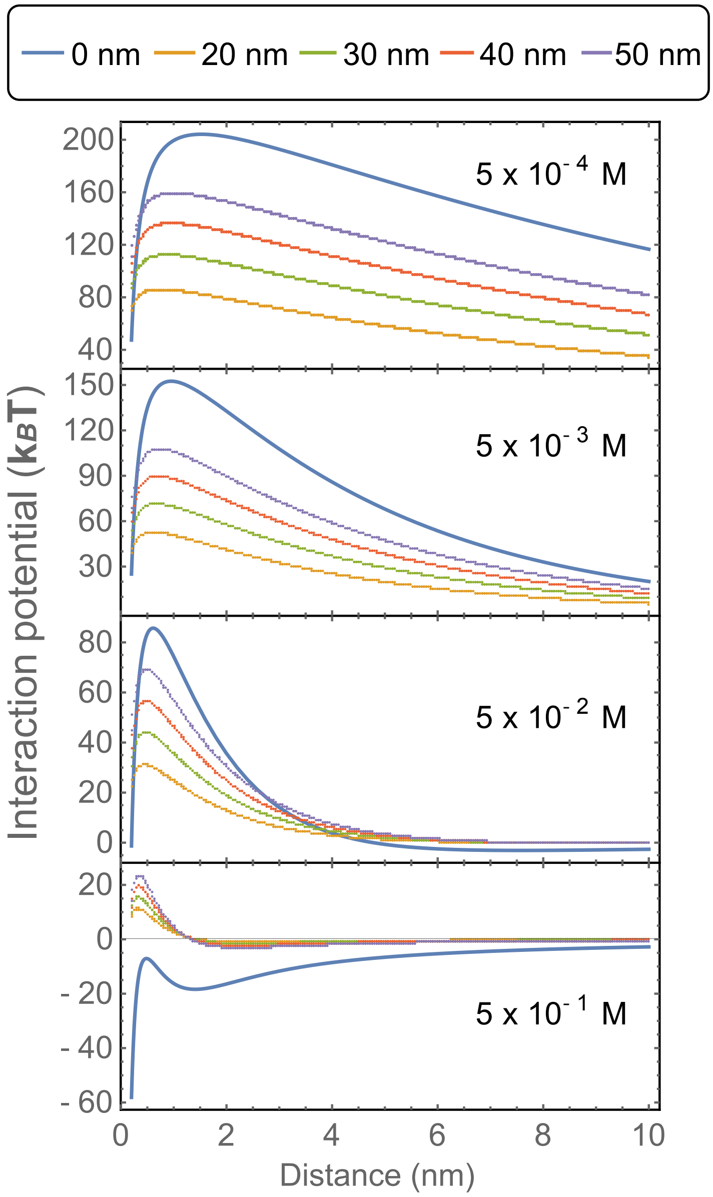

Supplementary Figure 12: Interaction potentials V_{DLVO} for raspberry colloids (b-v configuration) with different bump sizes but keeping the same overall size and for reference smooth PS particles (blue) at different salt concentrations of **a),** 0.0005 M, **b),** 0.005 M, **c)** 0.05 M and **d)** 0.5 M added monovalent salts. Only the b-v configuration is shown since it is more stable than the b-b configuration.

1.13 Effect of different salts

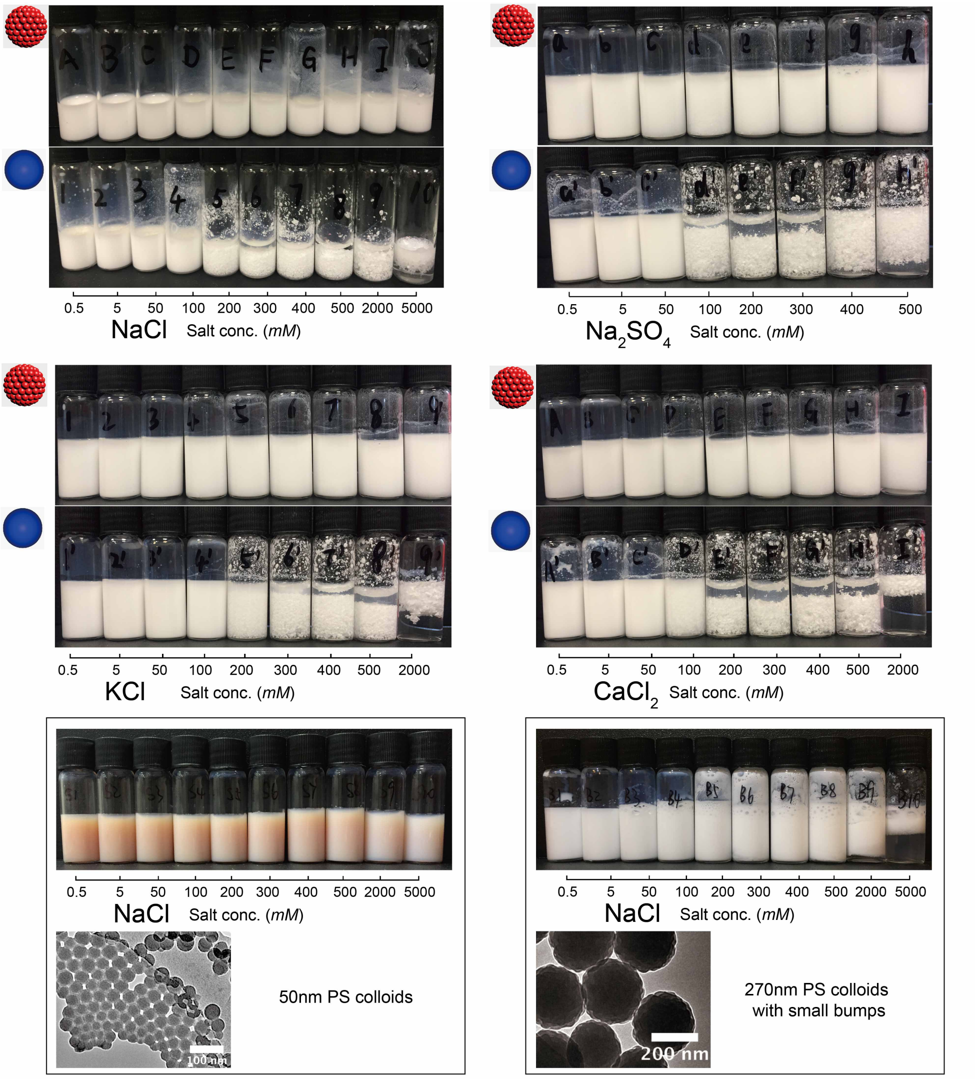

Supplementary Figure 13: Effect of different salts on the aggregation behaviour on raspberry, smooth PS, and the 'rough' PS particles – the latter are presented in Fig. S8a. The top four photographs show the effect on colloidal aggregation of raspberry and 280 nm large smooth PS particles caused by the 1:1 salts NaCl and KCl, as well as the 1:2 salt Na_2SO_4 and the 2:1 salt $CaCl_2$. The bottom left photograph shows the effect of adding NaCl to 50 nm large smooth PS particles, and the bottom right image illustrates the salting of aqueous solutions of larger 'rough' PS particles. The corresponding TEM images are presented as well. All samples had a colloid concentration of 3 w% and were sonicated for 1 minute before kept at room temperature.

1.14 Influence of zeta-potential on the colloidal stability

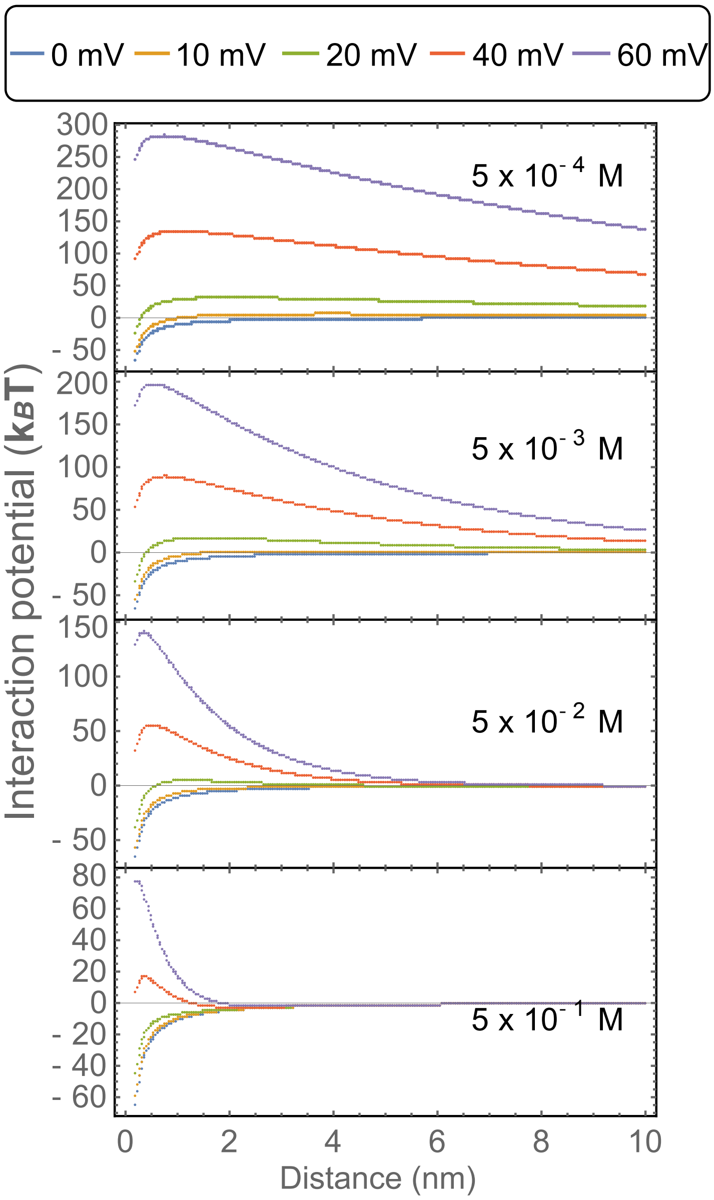

Supplementary Figure 14: Interaction potentials V_{DLVO} for the 280 nm large raspberry colloids with 45 nm bumps in the b-v configuration varying the zeta potentials ψ at different salt concentrations of **a),** 0.0005 M, **b),** 0.005 M, **c)** 0.05 M and **d)** 0.5 M added monovalent salts. Only the b-v configuration is shown since it more stable than the b-b configuration.

1.15 Colloidal stability of the other raspberry colloids

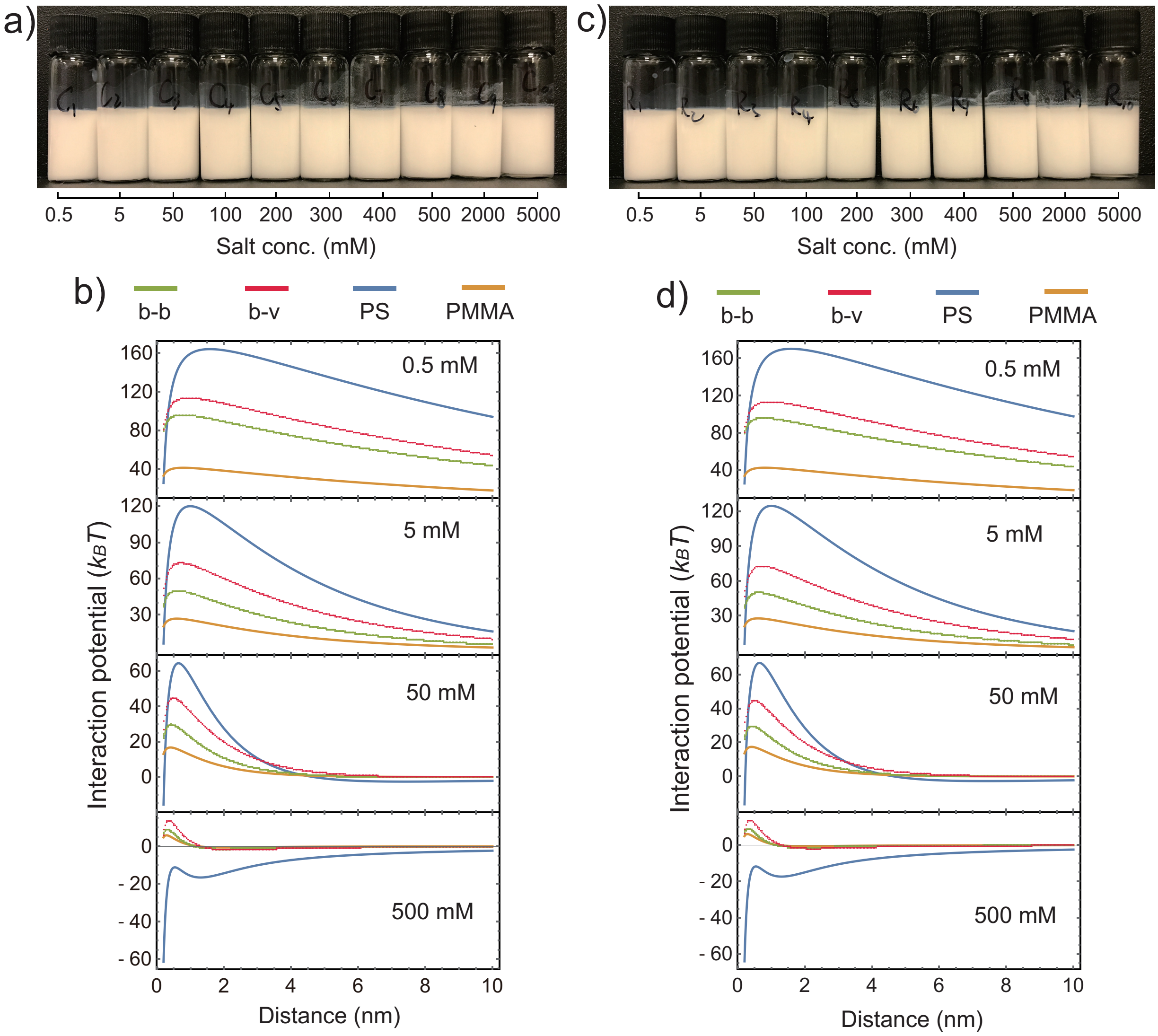

Supplementary Figure 15: a) Colloidal dispersion of Raspberry-MMA in water with different NaCl concentrations. b) Interaction potentials V_{DLVO} for Raspberry-MMA, smooth PS (245 nm) and nanoscale PMMA particles (35 nm) for 0.5 mM, 5 mM, 50 mM and 500 mM added NaCl. c) colloidal dispersion of Raspberry-EMA in water with different NaCl concentrations. b) Interaction potentials V_{DLVO} for Raspberry-EMA, smooth PS (255 nm) and nanoscale PMMA particles (35 nm) for 0.5 mM, 5 mM, 50 mM and 500 mM added NaCl.

2 Supplementary Tables

2.1 Table of the solubility of monomers

Entry	Monomer	Water (mM)	Mixture 1[b] (mM)	Mixture 2[c] (mM)
1	St	1.5	4	11
2	AM	55	150	195
3	DVB	<0.1	0.2	1.5

[a]The saturated solutions were all diluted 100 times before the UV-vis measurements. The solubility was obtained by fitting the absorption peak at respective wavenumbers (AM at 244 nm, St at 254 nm and DVB at 238 nm) in the calibration line; [b]Mixture 1 is water/ethanol mixture (80/20, v/v); [c]Mixture 2 is water/ethanol mixture (70/30, v/v).

Supplementary Table 1: Solubility of monomers in different continuous phases[a]

2.2 Table of the numerical coefficients

R_{PS}	140 nm
R_{PMMA}	22.5 nm
R_{PS}^{core}	117.5 nm
ψ	+42 mV
ν_e	3.0×10^{15} Hz
$n_{\mathrm{H_2O}}$	1.33
n_{PS}	1.557 [6]
n_{PMMA}	1.4905 [6]
$\varepsilon_{\mathrm{H_2O}}$	80 [6]
$\varepsilon_{\mathrm{PS}}$	2.55 [6]
$\varepsilon_{\mathrm{PMMA}}$	2.6 [6]

Supplementary Table 2: Numerical coefficients used in the model.

3 Supplementary Notes

3.1 Supplementary Note 1: Solubility of monomers

Key to successfully polymerising two different monomers into random copolymers that subsequently form composite colloids like the raspberries was the use of the right water-ethanol mixture as continuous solvent. To optimise this process we first tested the solubilities of the acrylic (AM) and styrene (St) monomers and the crosslinker divinylbenzene (DVB) in pure ethanol by measuring the UV-vis absorption for different monomer concentrations, providing us with a calibration curve shown in Supplementary Figure 1. These allow us to get an estimate of the different monomer solubilities from UV-vis absorption measurements in pure water, and two different water-ethanol mixtures (Supplementary Figure 2). While St monomers are hardly soluble in pure water the monomer solubility is enhanced by adding ethanol. Also the AM solubility is reduced in water but is clearly more enhanced in the presence of ethanol. The DVB crosslinker concentration is reduced the strongest in pure water. The 80/20 v/v water-ethanol mixtures, allowing to dissolve 4.0 mM, 0.2 mM and 150 mM of St, DVB and AM at room temperature respectively, delivers the most monodisperse and well-formed raspberry colloids (Fig. 2, Supplementary Table 1), as shown in section 1.3.

3.2 Supplementary Note 2: Effect of the continuous phase

Soap-free emulsion polymerisation (SFEP)[1, 2] and dispersion polymerisation (DP)[3] are simple and robust techniques to prepare colloidal particles. In SFEP,[1, 2] droplets of hydrophobic monomers are formed in water. After polymerisation sets in colloidal particles are budded off those emulsion droplets. In DP[3] the monomers and initiator are well soluble, forming a homogeneous solution initially. When polymerisation starts, the growing chains become increasingly insoluble and precipitate out of solution, eventually coagulating into colloidal particles. In the single-step preparation of raspberry colloids we combine both mechanisms: emulsion polymerisation sets in within the heterogeneous styrene-rich droplets (SFEP), in which some of the AM co-monomer and crosslinker are dissolved. Simultaneously, dispersion polymerisation of mainly acrylic monomers in the continuous phase (with small amounts of St and DVB) when the initiator is injected. Thus two different types of colloidal particles are formed in the continuous phase. These bind to each other through the cross-linker.

In deionised water the solubilities of the monomers decrease dramatically (1.5 mM, $<$0.1 mM and 55 mM for St, DVB and AM, respectively, Supplementary Table 1), resulting in a SFEP-like polymerisation. The resultant polymeric particles, shown in the TEM image in Fig. 2, are very polydisperse in size with an average diameter of 250 nm. Moreover, their surface is non-uniform but not quite as bumpy as the ideal raspberries formed in a continuous phase of water/ethanol (80/20 v/v) presented in Fig. 1. When a mixture of water/ethanol (70/30, v/v) was used as the continuous phase, the solubility of the monomers increased to 11 mM, 1.5 mM and 195 mM for St, DVB and AM, respectively (Supplementary Table 1). Raspberry colloids with an average core-diameter of 310 nm and an average diameter of the corona particles of 41 nm were obtained in addition to some randomly-distributed nanoscale particles (45 nm) as shown in Fig. 2c. These results indicate that the small colloidal particles were formed through dispersion polymerisation of the more soluble acrylic monomers, while the core of the raspberry colloids

were formed from the heterogeneous monomer droplets (as by SFEP).

3.3 Supplementary Note 3: Effect of the cross-linking agent

The presence of a cross-linking agent (DVB) and phase separation between PS and PA are also critical for the formation of raspberry colloids. When DVB was omitted from the synthesis of raspberry colloids, two main types of spherical particles (average diameter of 230 nm and 70 nm) are created as shown in TEM images (Fig. 2d). We identify the large particles as mainly PS colloids and the small ones as mainly PA colloids. The latter remained dispersed in solution rather than decorating the surfaces of the large PS particles [4].

We separated the small from the larger particles by centrifugation and performed FTIR and DLS measurements. The results indicate that the small particles contain much more PA segments than the larger particles (Supplementary Figure 5, indicated by the ellipse-shaped area with dashed border).

3.4 Supplementary Note 4: Effect of AM monomer

When the acrylate monomer (AM) was removed from the preparation of raspberry colloids in a 80/20 v/v water-ethanol mixture, spherical pure PS particles with an average diameter of 270 nm were observed (Fig. 2e,f). Small protrusions, visible as some surface roughness smaller than the 45 nm bumps in raspberries, are uniformly seen on all PS particles. We ascribe this surface roughness to the presence of ethanol that enhances the St-monomer solubility in the continuous phase, promoting emulsion and dispersion polymerisation. When we use half of the optimal AM-monomer concentration in the synthesis in a 80/20 v/v water-ethanol mixture, we obtain particles with irregular protrusions and non-spherical core shape as well as a large size distribution of the cores (Fig. 2f).

3.5 Supplementary Note 5: Other types of raspberry colloids

Based on the results discussed above, a new mechanism for preparation of raspberry colloids in a single step was proposed: This is based on exploiting the different solubilities of AM and St monomers in the water-ethanol mixture. Guided by this method, we prepared two additional raspberry colloids using different acrylate monomers instead of AM under the same conditions (Supplementary Figure 6). The average size (D_{TEM}) of the raspberry colloids prepared using methyl methacrylate (Raspberry-MMA, Supplementary Figure 6a) instead of AM was 245 nm in diameter with the small bumps on the surface being 35 nm in diameter. The average hydrodynamic diameter (D_{DLS}) of Raspberry-MMA measured by DLS is 250 nm (PDI=0.04). The (D_{TEM}) of the raspberry colloids prepared using ethyl methacrylate (Raspberry-EMA, Supplementary Figure 6b) instead of AM was 255 nm and the small bumps on the surface had a diameter of 35 nm. The D_{DLS} of Raspberry-EMA was 260 nm (PDI=0.07). The sizes of the small bumps are different because the formed acrylate polymers are of different hydrophobicity. The zeta-potential of the Raspberry-MMA and Raspberry-EMA were both +40 mV, measured in pure water.

3.6 Supplementary Note 6: Synthesis of PS particles

The 280 nm large PS colloids were synthesized by adding a monomer mixture of St (5.0 g, 48.0 mmol) and DVB (0.5 g, 3.8 mmol) to 100 mL deionised water. Then the initiator of 2,2′-azobis(2- methylpropionamide) dihydrochloride (AIBA, 187 mg, 0.7 mmol) was added to the mixture. Nitrogen was placed into the mixture for 1 h before elevating the temperature, and the nitrogen blanket was maintained throughout the polymerisation. The reaction was then left stirring at 80 °C for 24 h. The product was purified by dialysis against deionized water. The 50 nm large PS colloids were synthesized following the recipe described in ref. [5].

3.7 Supplementary Note 7: DLVO calculations

3.7.1 Model definition

We use the Derjaguin-Landau-Verwey-Overbeek (DLVO) theory to investigate the dramatic difference in colloidal stability and packing of raspberry and smooth particles. According to DLVO theory, the net interaction between two particles (V_{DLVO}) is the sum of the attractive van der Waals (V_{vdW}) and the repulsive electrostatic double-layer or Coulomb interactions (V_{Coul}) [6]:

$$V_{\text{DLVO}} = V_{\text{vdW}} + V_{\text{Coul}} \tag{1}$$

No three or other many-body interactions are considered.

We model a raspberry colloid as a particle composed of a spherical PS core and polymethylmethacrylate (PMMA) hemispheres grafted to the surface in a closely packed arrangement. PMMA was used instead of PA as all material parameters necessary for the model are known - though PMMA and PA are very similar. The PMMA radius is $R_{\text{PMMA}} = 22.5\ nm$ while the PS core radius is $R_{\text{PS}}^{\text{core}} = 117.5\ nm$. This totals to a raspberry-particle diameter of $D = 280\ nm$, as in the experiments. The PMMA hemispheres do not overlap. Furthermore, we assume that the PMMA hemispheres are stiff (hard spheres) and no additional steric interactions contribute to the total interaction energy. In order to obtain an approximate analytical formulation, we consider the total interaction as a sum of pair-wise interactions between PMMA hemispheres on different raspberries, plus the interactions between the PS core particles. We can then resort to analytical solutions of both V_{Coul} and V_{vdW} for spherical geometries.

3.7.2 Model Equations

The electrostatic interactions between two spheres of equal size and equal surface potential is calculated using the Poisson-Boltzmann formalism [6, 7] within the linearized form. They are given by:

$$V_{\text{Coul}} = \frac{\varepsilon_0 \varepsilon_r \kappa}{2} \left[\left(2\psi^2 \left(1 - \coth(\kappa h)\right) + 2\psi^2 \text{cosech}(\kappa h) \right) \right] \tag{2}$$

where ε_r is the relative dielectric constant of the solvent and h is the closest separation between the particle's surfaces[8]. ψ is the surface potential – we use the experimentally measured value $\psi = +42\,mV$. We measured for both raspberry and smooth PS particles almost the same values. κ is the Debye length,

given by:

$$\kappa = \sqrt{\frac{10^3 N_A e^2 c}{\varepsilon_0 \varepsilon_r k_\mathrm{B} T}}. \tag{3}$$

$N_A = 6.022 \times 10^{23}$ (Avogadro's Number), $e = 1.6 \times 10^{-19}$ C and c is the molar concentration of the electrolyte (here we used the monovalent 1:1 salt NaCl). The above equation is based on the Derjaguin approximation for calculating pair-wise interactions between spheres [9]. Since we consider only the closest hemisphere to the contact region between two interacting particles, already implicit in this approximation, the value of V_Coul calculated for spheres is adopted also for hemispheres. The electrostatic charge is assumed to be concentrated on the outer PMMA surfaces; only the last account for the total electrostatic interaction.

The van der Waals interactions between two spheres of equal size are calculated according to the Hamaker formalism of intermolecular forces, which assumes again pairwise additivity [7]. While this is known to be approximate, it nevertheless captures the physical origin of the interaction and the qualitative form of the potential [6]. The expression for the interaction potential is given by:

$$V_\mathrm{vdW} = -\frac{A_\mathrm{H}}{6}\left[\frac{2R^2}{h^2+4Rh} + \frac{2R^2}{h^2+4Rh+4R^2} + \ln\left(\frac{h^2+4Rh}{h^2+4Rh+4R^2}\right)\right] \tag{4}$$

where R is the radius of the particle and A_H is the Hamaker constant, which quantifies the material properties (interacting bodies and solvent), independently of the body's geometry [10]. The Hamaker constant is calculated on the basis of Lifshitz' theory of intermolecular forces [6]. For the symmetric case of two materials, 1 (the colloids) interacting across a medium 2 (the solvent)

$$A_\mathrm{H} = \frac{3}{4} k_\mathrm{B} T \left(\frac{\varepsilon_1 - \varepsilon_2}{\varepsilon_1 + \varepsilon_2}\right)^2 + \frac{3h\nu_e}{16\sqrt{2}} \frac{(n_1 - n_2)^2}{(n_1^2 + n_3^2)^{3/2}}. \tag{5}$$

ε_1 and ε_2 are the relative permittivities of the two materials and n_1 and n_2 are their refractive indices; ν_e is the main electronic absorption frequency in the UV and is typically around 3.0×10^{15} Hz; h is Planck's constant.

3.7.3 DLVO curves of single PS and PMMA particles

The DLVO potential curves calculated for two interacting pairs of single PS particles ($R_\mathrm{PS} = 140$ nm) and single PMMA spheres ($R_\mathrm{PMMA} = 22.5$ nm) for $c = 0.5$ mM are shown in Supplementary Figure 9. The numerical values are in good agreement with calculations performed on similar systems [11].

We stress that the electrolyte concentration affects V_Coul: a low value implies a long Debye screening-length κ and therefore longer-ranged repulsive forces.

It can be seen that the interaction between the PMMA spheres decays fast with the interparticle distance. Therefore, only the hemispheres in the contact region between two raspberry colloids actively contribute to the interaction potential (in terms of both V_vdW and V_Coul). The PS core particles are assumed to contribute only to the van der Waals interactions. The interactions between PS cores occurs through both the solvent and the PMMA hemispheres, which must be included in the calculation of the Hamaker constant

for the system of interest. According to the methodology adopted by Bahng et al. [12], the Hamaker constant for such a hybrid-dispersion medium is calculated as a weighted mean between the Hamaker constants of PMMA and solvent, with the weights being the volume fractions of the two media in the separation region. Supplementary Table 2 reports the different material properties implemented in the model. The solvent is water.

3.7.4 Raspberry colloids configurations

We investigated two typical configurations, depicted in Supplementary Figure 8: bump-to-bump (b-b) and bump-to-valley (b-v). In the b-b configuration, the sum of pair-wise interactions is assumed to depend only on the two couples of PMMA hemispheres closest to the contact region. In the b-v configuration, the sum of pair-wise interactions is assumed to involve only the four PMMA hemispheres closest to the contact region. In both configurations, opposite PMMA hemispheres that are not nearest neighbours are considered non-interacting, and the PS core particles contribute to the van der Waals interaction. The two potentials read as:

$$V_{\mathrm{DLVO}}^{b-b} = V_{\mathrm{vdW}}^{\mathrm{PS}} + \sum_{j=3}^{4} V_{\mathrm{DLVO}}^{1j} + \sum_{j=3}^{4} V_{\mathrm{DLVO}}^{2j} \tag{6}$$

$$V_{\mathrm{DLVO}}^{b-v} = V_{\mathrm{vdW}}^{\mathrm{PS}} + V_{\mathrm{DLVO}}^{13} + \sum_{j=3}^{4} V_{\mathrm{DLVO}}^{2j} \tag{7}$$

where V_{DLVO}, V_{VdW} and V_{Coul} are given by Equations (1-5) with appropriate parameters for the interacting pair in question, and the indices i and j indicate the interacting hemispheres, with reference to Supplementary Figure 8.

For each raspberry colloid it is possible to define an effective interaction layer as the flat PMMA layer with the same volume of the sum of the interacting hemispheres. The effective interaction plane is then defined as the boundary between the PMMA phase and the solvent phase. By definition, the distance between the effective interaction planes of two interacting raspberry particles will be always larger than the minimum distance between the hemispheres on the different colloids.

3.7.5 Results of DLVO calculations

Supplementary Figure 10 shows the different potential curves for the b-b, b-v, smooth PS and PMMA particles at different electrolyte concentrations. At low salt concentrations, the potential magnitude for both the raspberry configurations is smaller than for the PS particles but larger than for the PMMA particles. While one could expect a stronger attractive interaction due to the presence of additional contact points [13], the plots of the separate contributions show that the repulsive V_{Coul} has a longer range than V_{vdW} and dominates up to very short interparticle distances. This can be also explained in terms of the effective interaction plane defined in the previous section. Due to their roughness, the effective interaction planes for two interacting raspberry colloids are at a larger distance than for the smooth PS particles, thus resulting in a smaller overall repulsion. The fact that multiple couples of PMMA hemispheres participate in the interactions explains why the primary maximum for the raspberry colloids is larger than for the PMMA spheres.

At intermediate salt concentrations, the magnitude of the primary maximum drops for all the particle species and the interaction range shrinks due to the screening of the Coulomb repulsion. While the PS particles display a prominent V_{vdW} over V_{Coul}, the raspberry colloids show a smaller attraction potential due to their surface heterogeneity and V_{Coul} is strong enough to provide stabilisation. Moreover, V_{vdW} has a shorter range in comparison with the corresponding potential for the PS particles. The observation that surface roughness has the dual effect of both reducing attractive and repulsive interactions is consistent with previous findings [12, 14, 15].

At high salt concentrations, the energy barrier for the PS particles falls below 0 and they become unstable. The single PMMA particles show a low energy barrier of $\sim 5 k_{\mathrm{B}}T$, which can be easily overcome by thermal fluctuations, leading again to the instability of the dispersion. The situation for raspberry colloids is different – they still display a primary maximum of $\sim 15-20\ k_{\mathrm{B}}T$ which prevents their aggregation and confers stability. Again, this occurs since multiple couples of PMMA hemispheres participate in the interactions, leading to an overall sum of positive energy barriers and therefore to an enhanced primary maximum. The secondary minimum at a separation of $\sim 3\ nm$ is so shallow that although small clusters can form temporarily, they will fall apart due to thermal motion.

This explains the experimentally observed packing of the raspberries into photonic balls, while the smooth particles cannot achieve it due to the larger Coulomb repulsion, and subsequent instability upon drying of the droplet [16].

The colloid stability is further illustrated in Supplementary Figure 11, where the dependence of the maximum value in V_{DLVO} is plotted as function of added NaCl concentration for the different particles, and the separate contributions of V_{vdW} and V_{Coul} at this strongest repulsion. The maximum in V_{DLVO} for the smooth PS and both the raspberry particle configurations decreases gradually up to $c \simeq 5 \times 10^{-3}$ M. Upon further increasing the salt concentration, the maximum decreases more steeply for smooth PS colloids, disappearing at around $c_{cc} \simeq 0.3$ M, where c_{cc} indicates the critical coagulation concentration. In parallel, V_{vdW}, despite being small at low electrolyte concentration (even though it is larger than for the raspberry colloids), quickly decreases while the primary maximum position shifts towards a smaller distance and eventually overcomes V_{Coul} at c_{cc}. In contrast, the maximum for the raspberry colloids keeps gradually decreasing but never reaches the zero-level. There is no c_{cc} in the examined range, since V_{vdW} decreases less steeply than for smooth PS particles. We stress that the validity of the DLVO potential at higher ionic strength becomes questionable, due to the appearance of non-DLVO forces such as hydration forces [17, 18, 19], finite-size ion effects [20] and specific ion effects known as Hofmeister effects [21].

3.7.6 Influence of bump-size on the colloidal stability

We further investigated the effect of the size of the bumps on the colloidal stability. Supplementary Figure 12 compares the DLVO curves of the raspberry particles (b-v configuration) with four different bump sizes and the smooth PS particles with the same overall diameter. In general, all the rapsberry particles are found to exhibit a similar behavior to the one described in the main text, namely less overall repulsion than their smooth PS counterpart at low salt concentrations and better stability at higher salt concentrations. Moreover, the configurations with larger bumps tend to display stronger electrostatic repulsion. This is expected since the distance between the effective interaction planes between two raspberry colloids increases with increasing the bump size, leading to smaller effective van der Waals attractions. As a

consequence, raspberries with larger bumps are more stable at high salt concentrations. This suggests that the size of the bumps plays an important role in the colloidal stability. We stress that the current model limits the validity of these observations to bumps larger than 20 nm and smaller than 60 nm. If the bumps are smaller than 20 nm more than two couples of hemispheres would be interacting, while if the bumps are larger than 60 nm the curvature of the core particle would need to be taken into account.

3.7.7 Influence of zeta-potential on the colloidal stability

We also investigated the effect of the zeta potential ψ of the raspberry particles on the colloidal stability. Supplementary Figure 14 compares the DLVO curves of the 280 nm large raspberry particles (45 nm bumps) with five different zeta potential values, ranging from 0 to a maximum of 60 mV which we typically can achieve in many colloidal preparations. Moreover, zeta potentials larger than that are not well described by standard DLVO theory, as already mentioned. We observe that the raspberries display aggregation at large salt concentrations for ψ smaller than 20 mV while they are stable for larger potential values. This suggests that morphology alone is not sufficient to provide stabilization and a mild electrostatic repulsion is necessary to counterbalance the van der Waals attraction. We note, though, that while non-charged raspberries (blue curve) display large van der Waals attractions already at low salt concentrations, even small zeta potentials are enough to stabilize the raspberry particles at almost all NaCl concentrations. This further confirms that well-controlled protrusions plays a crucial role in greatly enhancing the stabilization of colloidal particles.

3.7.8 Colloidal stability of the other raspberry colloids

We also tested the stability of the other two raspberries prepared using the single-step method in water with different added NaCl concentrations. Supplementary Figure 15a and 15c show the photographs of salting series of Raspberry-MMA and Raspberry-EMA aqueous dispersions, respectively. It shows that, similar to the raspberry colloids made by AM, Raspberry-MMA and Raspberry-EMA can also stably dispersed in water even with high added NaCl salt (5000 mM, Supplementary Figure 15a and 15c). The corresponding interaction potentials V_{DLVO} for Raspberry-MMA and Raspberry-EMA for 0.5 mM, 5 mM, 50 mM and 500 mM added NaCl were shown in Supplementary Figure 15b and 15d, respectively.

3.8 Supplementary Note 8: Structural color

The organisation of raspberry colloids into a regular FCC packing provides the typical response of opalescent photonic crystals [22]. By approximating the refractive index of the raspberries with the one of polystyrene ($n = 1.57$) and using as radius $a = 143$ nm, we observe that the reflection in the red, shown in Fig. 4a of the main text, corresponds to the stop-band in the $\Gamma - L$ direction. Supplementary Figure 7 shows the band diagram for an FCC geometry calculated using the freely available software package MIT Photonic-Bands (MPB) [23]. Similar to opals build of spherical particles and made of a single material, it is possible to tune the reflected colour by changing the size of the raspberry colloids.

In case of a planar geometry, by illuminating the sample with a low numerical aperture source (NA=0.1) we mainly probe the sample in the $\Gamma - L$ direction. Therefore we observe a red uniform reflection from the sample, as seen in Fig. 4a. However, when analysing the optical response from the raspberry-superballs

it is necessary to consider the spherical geometry of the system as shown in Figure 4b. This is similar to previously reported systems[24, 25].

The red reflection spot seen in the middle of the raspberry-superspheres (Fig. 4b) is the result of the back reflection from the top of the sphere. The dimension of the spot depends on the dimension of the numerical aperture of the illumination, while the numerical aperture of collection is fixed by the objective (NA=0.6). Similarly the light streaks at the position where the raspberry-superspheres are in contact (Fig. 4b) are caused by reflected rays bouncing back after having been reflected from one supersphere to another. This is schematically described in the top part of Fig. 4 and happens when the surface normal of the sphere is at 45° to the incident beam. By geometrical considerations, this appears at a distance of $r_1 = R\sin 45^\circ = R/\sqrt{2}$ from the centre, where R is the radius of the supersphere.

3.9 Supplementary Note 9: Effect of different salts on the stability of raspberry dispersion

We tested the stability of the raspberry and smooth PS-particle suspensions also for different salts. Both particle species are roughly 280 nm large and have a similarly positively charged surface. In Supplementary Figure 13 we show photographs with salting series for two different 1:1 salts (NaCl and KCl) as well as a 2:1 salt ($CaCl_2$) and a 1:2 salt (Na_2SO_4). In all cases the raspberry-colloid samples remained well dispersed even for high added salt concentrations. Note that in some cases added salt concentrations did not exceed 0.5 M because of the solubility limit of the salt used. For all three samples with Cl^- as monovalent anion the screening of the Coulomb interactions was similar leading to flocculation of the smooth PS particles at around 100 mM. As the Debye screening length for the SO_4^{-2} anion is stronger than the monovalent Cl^- flocculation sets in at lower added salt concentrations. Testing our hypothesis that the actual size and thus the strength of the van der Waals attraction between particles is important, we also tested the salting effect on smooth 50 nm large PS colloids. These contained a different monomer making the solutions appear reddish, but their zeta-potential was equivalent to that of the larger PS particles. Note that the atomic polarizabilities of PS and PMMA are very similar in value and therefore have a similar Hamaker constant. These small particles also remain stable in high-salt conditions similar to the larger raspberry colloids (Supplementary Figure 13, bottom left). Further we tested the effect of 'roughness' on the colloidal stability by preparing a similar salting series on the rough 270 nm large PS particles presented in Figure 2e. In Supplementary Figure 13, bottom right, it becomes apparent that the roughness increases the PS-particles' stability, suggesting that only a large enough roughness will bring about the stability observed in raspberry-particle suspensions. Above 250 mM added NaCl there seems to be an onset of aggregation and at 2 M added NaCl clear phase separation sets in simply because of the increased density of the salty aqueous phase.

References

[1] Chern, C. Emulsion polymerization mechanisms and kinetics. *Prog. Polym. Sci.* **31**, 443 – 486 (2006).

[2] Rao, J. P. & Geckeler, K. E. Polymer nanoparticles: Preparation techniques and size-control parameters. *Prog. Polym. Sci.* **36**, 887–913 (2011).

[3] Richez, A. P., Yow, H. N., Biggs, S. & Cayre, O. J. Dispersion polymerization in non-polar solvent: Evolution toward emerging applications. *Prog. Polym. Sci.* **38**, 897–931 (2013).

[4] Kraft, D. J. *et al.* Surface roughness directed self-assembly of patchy particles into colloidal micelles. *Proc. Natl Acad. Sci. USA* **109**, 10787–10792 (2012).

[5] Lan, Y., Wu, Y., Karas, A. & Scherman, O. A. Photoresponsive hybrid raspberry-like colloids based on cucurbit[8]uril host-guest interactions. *Angew. Chem. Int. Ed.* **53**, 2166–2169 (2014).

[6] Israelachvili, J. N. *Intermolecular and Surface Forces* (Academic Press, 2011), 3rd edn.

[7] Russel, W. B., Saville, D. A. & Schowalter, W. R. *Colloidal Dispersions* (Cambridge University Press, Cambridge, 1992).

[8] Hogg, R., Healy, T. W. & Fuerstenau, D. W. Mutual coagulation of colloidal dispersions. *Trans. Faraday Soc.* **62**, 1638 (1966).

[9] Derjaguin, B. Untersuchungen über die Reibung und Adhäsion, IV. *Kolloid-Zeitschrift* **69**, 155–164 (1934).

[10] Elimelech, M. *et al.* Surface interaction potentials. In *Particle Deposition & Aggregation*, 33–67 (1995).

[11] Liao, Q., Chen, L., Qu, X. & Jin, X. Brownian Dynamics Simulation of Film Formation of Mixed Polymer Latex in the Water Evaporation Stage. *J. Colloid Interface Sci.* **227**, 84–94 (2000).

[12] Bahng, J. H. *et al.* Anomalous dispersions of 'hedgehog' particles. *Nature* **517**, 596–599 (2015).

[13] Delrio, F. W. *et al.* The role of van der Waals forces in adhesion of micromachined surfaces. *Nat. Mater.* **4**, 629–634 (2005).

[14] Huang, X., Bhattacharjee, S. & Hoek, E. M. V. Is Surface Roughness a "Scapegoat" or a Primary Factor When Defining Particle-Substrate Interactions? *Langmuir* **26**, 2528–2537 (2010).

[15] Bhattacharjee, S., Ko, C.-H. & Elimelech, M. DLVO Interaction between Rough Surfaces. *Langmuir* **14**, 3365–3375 (1998).

[16] Fong, C. S., Black, N. D., Kiefer, P. a. & Shaw, R. a. An experiment on the Rayleigh instability of charged liquid drops. *Am. J. Phys.* **75**, 499–503 (2007).

[17] Pashley, R. Hydration forces between mica surfaces in aqueous electrolyte solutions. *J. Colloid Interface Sci.* **80**, 153–162 (1981).

[18] Horn, R., Smith, D. & Haller, W. Surface forces and viscosity of water measured between silica sheets. *Chem. Phys. Lett.* **162**, 404–408 (1989).

[19] Marčelja, S. Exact Description of Aqueous Electrical Double Layers. *Langmuir* **16**, 6081–6083 (2000).

[20] Vigil, G., Xu, Z., Steinberg, S. & Israelachvili, J. Interactions of Silica Surfaces. *J. Colloid Interface Sci.* **165**, 367–385 (1994).

[21] Parsons, D. F., Boström, M., Lo Nostro, P. & Ninham, B. W. Hofmeister effects: interplay of hydration, nonelectrostatic potentials, and ion size. *Phys. Chem. Chem. Phys.* **13**, 12352–12367 (2011).

[22] Joannopoulos, J. D., Johnson, S. G., Winn, J. N. & Meade, R. D. *Photonic Crystals: Molding the Flow of Light* (Princeton Unviersity Press, 2011), 2nd edn.

[23] Johnson, S. & Joannopoulos, J. Block-iterative frequency-domain methods for Maxwell's equations in a planewave basis. *Opt. Express* **8**, 173 (2001).

[24] Vogel, N. *et al.* Color from hierarchy: Diverse optical properties of micron-sized spherical colloidal assemblies. *Proc. Natl Acad. Sci. USA* **112**, 10845–10850 (2015).

[25] Noh, J., Liang, H.-L., Drevensek-Olenik, I. & Lagerwall, J. P. F. Tuneable multicoloured patterns from photonic cross-communication between cholesteric liquid crystal droplets. *J. Mater. Chem. C* **2**, 806–810 (2014).
