# Supplementary material for: Unexpected stability of aqueous dispersions of raspberry-like colloids
Source: Nat Commun. 2018 Sep 6;9:3614. doi: 10.1038/s41467-018-05560-3 (PMC6127241; doi:10.1038/s41467-018-05560-3)
Supplement: Supplementary file 2 — Description of Additional Supplementary Files [file 41467_2018_5560_MOESM2_ESM.pdf]

Description of Additional Supplementary Files

File Name: Supplementary Movie 1
Description: TEM images rendered into a video demonstrating the 3D structure of a raspberry colloid.

File Name: Supplementary Movie 2
Description: Microscopy video showing the drying of emulsion droplets containing smooth, 280 nm large PS colloids - below a critical size these droplets disintegrate.
